# Convergent 3–6 kHz cochlear vulnerability in aircrew: matching threshold sound conditioning targets to operational flight profiles

**DOI:** 10.3389/fneur.2026.1903374

**Published:** 2026-07-28

**Authors:** Daehee Lee, Sungshin Jang, Ku Kang, Eunjeong Choi, Chan-Mo Yang, Jin Yoo, Soohwan Kim, Sangyeop Kwak, Doo-Hee Lee

**Affiliations:** 1 Sound Vaccine, Inc., Daejeon, Republic of Korea; 2 Goldenear Company, Inc., Valencia, CA, United States; 3 Chemical, Biological, Radiological and Nuclear (CBRN) Defense Research Institute, Ministry of National Defense, Seoul, Republic of Korea; 4 Independent Researcher, Seoul, Republic of Korea; 5 Department of Psychiatry, Wonkwang University School of Medicine, Iksan, Republic of Korea; 6 AudioCardio, Inc., Santa Monica, CA, United States

**Keywords:** aircrew, cochlear vulnerability, cosmic radiation, hypobaric hypoxia, machine-learning, sensorineural hearing loss, threshold sound conditioning

## Abstract

Commercial and military aircrew operate within a multi-hazard auditory environment that combines cabin acoustic noise, chronic hypobaric hypoxia under regulatory cabin-pressure caps, and altitude-dependent cosmic ionizing radiation. Their convergent action on cochlear vulnerability has not been quantified, and no decision framework exists for matching threshold sound conditioning (TSC) target frequencies to operational flight profiles. This work is a computational, hypothesis-generating framework rather than an empirical study. We propose that the dominant frequency band of cochlear vulnerability in aircrew (3–6 kHz) emerges as the geometric overlap of three mechanism-distinct vulnerability kernels and coincides with the TSC selection region (3, 4 or 6 kHz) evaluated in the Kwak and Kwak 2020 randomized controlled trial. We construct a Cumulative Cochlear Stress Index (CCSI) that combines a CARI-7-derived radiation dose formula, an estimated-alveolar-tension hypoxia term and an A-weighted noise term, weighted by frequency-resolved Gaussian vulnerability kernels in log-octave space. The geometric mean of the three kernels peaks at f^*^ = 4.23 kHz (FWHM 2.06–8.72 kHz) and is consistent with the convergence hypothesis on the discrete clinical TSC grid (argmax 4 kHz). A sensitivity analysis over the full audiometric grid {0.5, 1, 2, 3, 4, 6, 8, 12} kHz, six mixing-weight scenarios (including a radiation-excluded *w*_*R*_ = 0 case) and four aircrew profiles finds the global CCSI argmax inside 3/4/6 kHz in all 24 combinations, and a Monte-Carlo randomization of the kernel parameters within literature bounds reproduces the 4 kHz convergence in 99.2% of draws, vs. 23.1% for unconstrained kernels, a non-tautological robustness check. Acoustic noise and hypobaric hypoxia are treated as the primary, mechanistically supported axes, while cosmic ionizing radiation is retained as an *exploratory* third axis given the absence of direct cochlear evidence at cruise-altitude doses. A machine-learning identifiability check (Random Forest, Gradient Boosting and Ridge ensembles on a 500-profile synthetic dataset; band-averaged *R*^2^≈0.98) is reported as an internal model-recovery check rather than a biological validation. The framework supports prospective evaluation of TSC in aircrew cohorts.

## 1 Introduction

### 1.1 Aircrew as a cochlear-risk population

Pilots and cabin attendants represent one of the longest-studied occupational populations with documented sensorineural hearing loss (SNHL). A cross-sectional audiometric study of *n* = 3, 130 Brazilian civilian pilots found a noise-induced hearing loss prevalence of 18.9% at 4 kHz, 15.8% at 6 kHz and 9.5% at 3 kHz, with the classical noise-notch pattern centered near 4 kHz (1). The same pattern is reproduced in fighter-pilot cohorts using extended high-frequency audiometry, where significant threshold elevation in the 4–8 kHz band emerges relative to age-matched controls (2), in Swedish commercial pilot and cabin-crew surveys (3, 4), in helicopter pilots vs. air-traffic controllers (5) and in larger Saudi military cohorts (6). After-flight temporary threshold shifts are recoverable but cumulative; extended high-frequency audiometry detects the earliest audiometric signal of damage before conventional pure-tone audiometry becomes positive (7, 8). Cabin acoustic measurements compiled by the U.S. Government Accountability Office report cabin LA_*eq*_ values of 74–84 dBA, cockpit values of 75–81 dBA, and military/fighter cockpit values reaching 85–95 dBA (9).

### 1.2 Beyond noise: a multi-hazard cochlear environment

What is less appreciated in audiology is that aircrew do not operate under noise alone. At cruise altitude they sustain two additional physiologically relevant cochlear stressors that operate continuously over each flight segment: chronic hypobaric hypoxia under the regulatory cabin-pressure schedule, and a level of cosmic ionizing radiation that rises exponentially with altitude through the residual atmosphere. Although each of these has been studied in isolation, their convergent action on the cochlea has not been treated quantitatively in the audiology literature.

### 1.3 Hypobaric hypoxia and the cochlea

Federal aviation regulation 14 CFR 25.841 caps the cabin altitude during cruise at ≈ 2.44 km (8,000 ft equivalent), but this still drives the estimated alveolar oxygen tension *P*_*A*_*O*_2_ from ≈ 99 mmHg at sea level to ≈ 55 mmHg at the cap (a ≈ 45% reduction under the simplified West alveolar gas equation; see Equation 6). Arterial *P*_*a*_*O*_2_ is somewhat lower than *P*_*A*_*O*_2_ due to the A–a gradient, and we use *P*_*A*_*O*_2_ throughout as a proxy for arterial oxygen availability. This reduced alveolar tension is sustained for the duration of the flight segment. The ADEMED 2011 high-altitude expedition demonstrated in volunteers acclimatized over 10 days at 5,300 m that the same noise dose produces a substantially larger temporary threshold shift under hypobaric hypoxia than at sea level, and that acclimatization does *not* protect the inner ear despite raising arterial oxygen saturation (10). A parallel 1-year cohort of soldiers permanently stationed at high altitude reported persistent threshold elevation centered at 2–4 kHz (11), and a rat auditory-brainstem-response model demonstrated synergistic noise–hypoxia susceptibility of the cochlea (12). Aviation otology additionally recognizes middle-ear barotrauma at descent as a peripheral mechanism that compounds inner-ear stress in the same flight segment (13–17).

### 1.4 Cosmic ionizing radiation, with limited cochlear evidence

Aircrew are classified as an occupationally exposed population under *ICRP Publication 103* (18), and large multi-national epidemiological cohorts confirm elevated incidence of specific malignancies that co-vary with cumulative cosmic-radiation dose (19–22), with cosmic ionizing radiation increasingly characterized as a DNA-damaging agent in flight crews (23). The galactic cosmic-ray flux at cruise altitude is modeled by the U.S. FAA CARI-7 dose model (24) and the European NAIRAS framework (25, 26), and in-flight Liulin spectrometry over the Korean peninsula at FL300 (9, 144 m) reports ambient dose-equivalent rates of 2.3 ± 0.17μSv h^−1^ (27). A simple exponential altitude–dose approximation to CARI-7 outputs under Solar Cycle 25 maximum conditions (used here as a screening-level dose proxy; see Section 2.4 and Equations 1, 5) implies annualized effective doses at ~ 40,000 ft routinely exceed the ICRP public reference level of 1 mSv yr^−1^ and reach 1.7–2.2 mSv yr^−1^ on polar routes.

A direct, dose-matched epidemiological link between the cruise-altitude cosmic-radiation environment and human cochlear dysfunction is *not currently available*. The only mechanistic evidence comes from clinical head-and-neck radiotherapy at far higher cochlear doses (35–70 Gy): a dose-response transition near 35–45 Gy (28), a high-frequency-biased prevalence pattern (29), and ribbon-synapse disruption (30), which we review quantitatively in Section 2.5 rather than repeat here. We therefore treat ionizing radiation as a *candidate*, frequency-resolved contributor rather than an established aviation auditory hazard.

### 1.5 A convergent 3–6 kHz vulnerability band

The three pathways above place their cochlear emphasis on overlapping, but not identical, frequency bands. The classical noise-notch peaks near 4 kHz with shoulders at 3 and 6 kHz (1). The hypoxia-conditioned threshold-shift band peaks at 2–4 kHz (10, 11), shifted toward lower-mid frequencies relative to pure noise. The radiation-cochlea band is high-frequency-biased and peaks at 6–8 kHz, reflecting basal ribbon-synapse vulnerability (29, 30). The geometric overlap of these three bands defines a 3–6 kHz region that is the central concern of the present paper.

### 1.6 Threshold sound conditioning as a frequency-selective therapeutic paradigm

Within otology, threshold sound conditioning (TSC) has been clinically evaluated in a randomized controlled trial as a paradigm for inducing adult auditory pathway plasticity. The principle draws on the seminal Noreña and Eggermont demonstration that an enriched acoustic environment at frequencies matched to the deprived cochlear region prevents post-trauma reorganization of the primary auditory cortex tonotopic map (31–34). Kwak & Kwak operationalized this principle into a clinical protocol that delivers a sub-threshold tone, individualized to the patient's worst-threshold frequency *among 3, 4, or 6 kHz*, for 1 h per day over two to 3 weeks via a behind-the-ear hearing-aid platform. In a Stanford-IRB-approved double-blind randomized controlled trial of 39 evaluable subjects, the TSC arm achieved threshold amelioration in 78% of participants vs. 44% in the control arm [*p* = 0.008 (35–37)]. The protocol's empirical choice of 3, 4 or 6 kHz as the candidate conditioning band is exactly the convergence region identified above.

### 1.7 Knowledge gap and contributions

What remains absent from the literature is a quantitative framework that connects the multi-hazard operational environment of aircrew to the frequency-resolved TSC target. The clinical TSC protocol selects the conditioning frequency from the patient's worst pure-tone threshold, but in a preventive aviation-medicine context, where audiometric loss has not yet developed but cumulative exposure already has, the prioritization must instead be derived from the flight profile itself (i.e., *a priori*, before any audiogram is available).

The present study advances four contributions.

1. We synthesize the available aviation-medicine, hypoxic-cochlea, ionizing-radiation and TSC literature into an explicit *3–6 kHz convergence hypothesis*, identifying the frequency band at which cabin-noise and hypobaric-hypoxia vulnerability co-localize on the cochlea, with cosmic ionizing radiation considered as an *exploratory* high-frequency-biased third axis rather than an established hazard (Sections 2–4).
2. We introduce the Cumulative Cochlear Stress Index (CCSI), a physics- and audiology-informed predictive model that takes flight altitude, geomagnetic cut-off rigidity, heliocentric potential, flight-hours and cabin acoustic level as inputs and returns a frequency-resolved vulnerability score across eight standard audiometric bands (Section 5). The radiation component of CCSI is anchored to a screening-level approximation to the CARI-7 dose model (24) and is benchmarked against published in-flight aircrew dosimetry (27, 38, 39), and the frequency kernels are calibrated from published audiometric and mechanistic data.
3. We provide a machine-learning *identifiability check* [Random Forest (40), Gradient Boosting (41) and Ridge ensembles, implemented in scikit-learn (42), on a 500-profile synthetic dataset] confirming that each operational input activates the frequency bands predicted by its underlying physiology. Because the regression labels are themselves CCSI values, this is an internal model-recovery check, not a biological validation, and is reported in full in Supplementary material.
4. We apply the framework to four representative aircrew operational profiles (polar long-haul, mid-latitude long-haul, domestic short-haul, and military fighter/rotary) and report the recommended TSC target frequency within the clinical 3/4/6 kHz set defined by the Kwak & Kwak trial (Section 6).

The framework is intended as a transparent decision-support tool that supports prospective evaluation of frequency-targeted TSC in aircrew cohorts, in advance of the audiometric data required to test the hypothesis directly.

## 2 Multi-hazard auditory environment at cruise altitude

**Figure 1:**
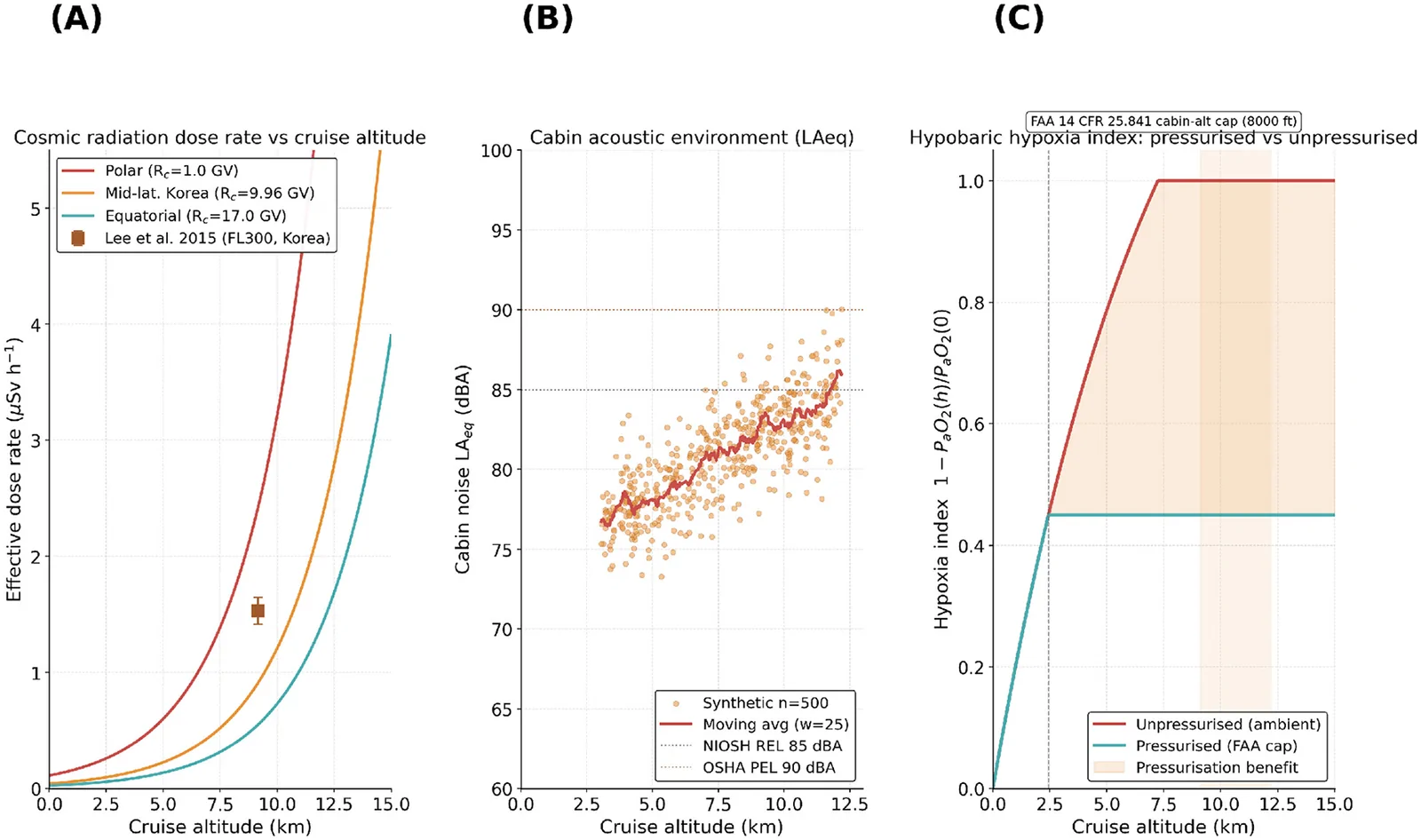
Multi-hazard auditory environment of cruise-altitude operations. **(A)** Effective dose rate of galactic cosmic radiation vs. cruise altitude under Solar Cycle 25 maximum conditions, parameterized by geomagnetic cut-off rigidity *R*_*c*_. Square marker: Lee et al. (27) in-flight Liulin measurement at FL300 over the Korean peninsula (converted from H^*^(10) to effective dose). **(B)** Cabin acoustic environment (LA_*eq*_) vs. cruise altitude from a synthetic (*n* = 500) dataset, with NIOSH REL 85 dBA and OSHA PEL 90 dBA reference levels. **(C)** Hypobaric hypoxia index 1−*P*_*A*_*O*_2_(*h*)/*P*_*A*_*O*_2_(0) alveolar oxygen tension under the simplified West alveolar gas equation; see Equation (6), contrasting unpressurized exposure with the FAA 14 CFR 25.841 pressurization cap (cabin altitude bounded at ≈2.44 km).

This section consolidates the quantitative characteristics of the three principal cochlear stressors acting simultaneously during commercial and military flight operations, and the limited but mechanistically suggestive evidence linking cruise-altitude cosmic radiation to cochlear damage. The combined picture is summarized in Figure 1.

### 2.1 Cabin and cockpit acoustic environment

The acoustic level inside a commercial aircraft cabin during cruise is determined by engine and aerodynamic noise transmitted through the fuselage. Compiled measurements summarized by the U.S. Government Accountability Office report cabin LA_*eq*_ values of 74–84 dBA on typical wide-body and narrow-body cruise segments, cockpit values of 75–81 dBA, and military or fighter cockpit values reaching 85–95 dBA, with peak A-weighted excursions during cabin-call signals and turbulence reaching 105 dBA (9). In our synthetic dataset (Figure 1B; *n* = 500 uniformly sampled profiles between 3 and 12 km), the implied cabin LA_*eq*_ trends modestly upward with cruise altitude, reflecting both higher engine thrust and the lower density-driven aerodynamic excitation of the fuselage.

**Figure 2:**
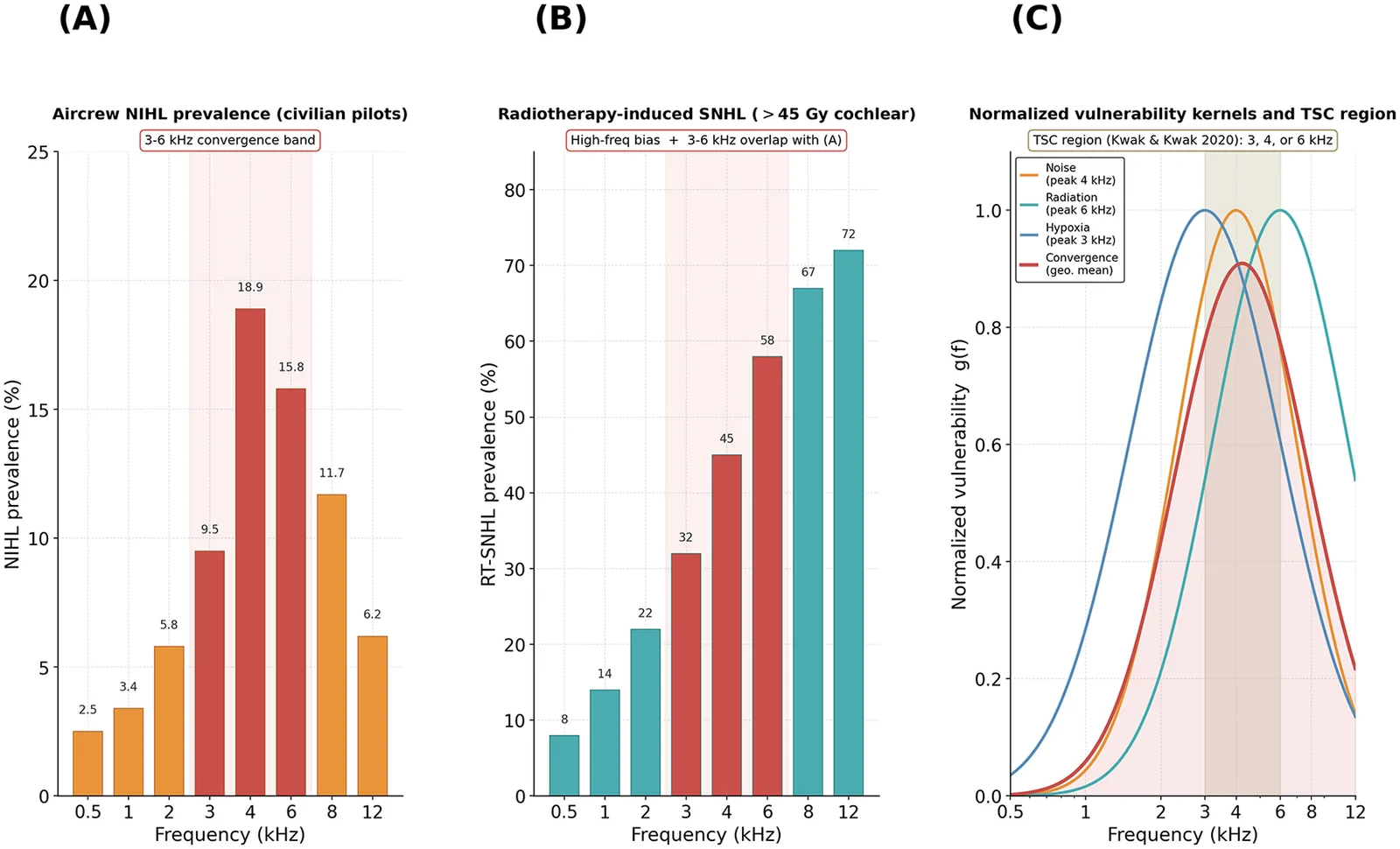
Convergent 3–6 kHz cochlear vulnerability across noise, radiation and hypoxia pathways. **(A)** Aircrew NIHL prevalence per audiometric band from Falcão et al. 2014 (*n* = 3, 130 civilian pilots) (1). **(B)** Radiotherapy-induced SNHL prevalence per audiometric band for cochlear doses >45 Gy (28, 29). **(C)** Normalized vulnerability kernels for noise (Gaussian peak 4 kHz), radiation (peak 6 kHz) and hypoxia (peak 3 kHz) in log-octave frequency space, with the geometric-mean *convergence* curve and the clinical TSC region [3, 4 or 6 kHz; (35)].

Although these LA_*eq*_ values mostly remain below the OSHA permissible exposure limit of 90 dBA for an 8-h reference shift and only marginally exceed the more conservative NIOSH recommended exposure limit of 85 dBA, two operational features of aviation are not adequately captured by the standard time-weighted average. First, cumulative exposure for long-haul wide-body aircrew, who routinely log 600–900 flight-hours per year, integrates to a substantially higher annualized dose than the single-shift OSHA reference assumes. Second, the frequency content of cabin and cockpit noise is concentrated in the 0.5–4 kHz band, overlapping the cochlear regions of highest mechanical vulnerability and producing the classical 4 kHz noise-notch documented in the Brazilian (*n* = 3, 130; Figure 2A) (1) and military (2–6) cohorts cited in Section 1.

### 2.2 Hypobaric hypoxia under regulatory cabin pressurization

Federal aviation regulation 14 CFR 25.841 mandates that the cabin altitude be maintained at or below ≈ 2.44 km (8,000 ft equivalent) during cruise. Applying a simplified alveolar gas equation under standard barometric assumptions ($P_{\text{atm}}\left(h\right)=760\cdot e^{-h/7.4}$ mmHg, *F*_*i*_*O*_2_ = 0.2095, *P*_*a*_*CO*_2_ = 40 mmHg, RQ = 0.8) yields an estimated alveolar oxygen tension *P*_*A*_*O*_2_ that falls from ≈ 99 mmHg at sea level to ≈ 55 mmHg at the regulatory cap, a ≈ 45% reduction. The strict alveolar gas equation returns *P*_*A*_*O*_2_, not arterial *P*_*a*_*O*_2_; the two differ by an A–a gradient that depends on ventilation–perfusion matching and individual physiology, and we therefore use *P*_*A*_*O*_2_ as a proxy for arterial oxygen availability throughout. The implied alveolar hypoxia index *H*(*h*) = 1−*P*_*A*_*O*_2_(*h*)/*P*_*A*_*O*_2_(0) is plotted in Figure 1C; pressurization flattens what would otherwise be a monotonically rising exposure into a plateau, but the plateau itself is non-negligible and is sustained for the entire cruise segment.

The biological relevance of this residual hypoxia for the cochlea is established by the ADEMED 2011 high-altitude expedition. After 10 days of acclimatization at 5,300 m (Gorak Shep, Solo Khumbu, Nepal), the same 90 dB white-noise exposure produced a substantially larger temporary threshold shift than at low altitude; critically, acclimatization did not protect the inner ear, although it did raise arterial oxygen saturation (10). The mechanism, supported by complementary auditory-brainstem-response work in rats (12), is thought to reflect oxygen-supply limitations at the stria vascularis under the combined demand of acoustic stimulation, with insufficient mitochondrial support of the active hair-cell amplifier in basal-cochlear regions. A 1-year longitudinal cohort of soldiers permanently stationed at high altitude reported persistent threshold elevation centered at 2–4 kHz (11), the frequency band that geometrically overlaps with the noise-notch peak.

### 2.3 Otitic barotrauma at descent

Cabin pressurization also exposes the middle ear to repeated pressure gradients during descent, with implications that are peripheral but not negligible for inner-ear stress. Otic barotrauma during air travel arises from inadequate equalization between middle-ear and external atmospheric pressure across the Eustachian tube (13, 15–17). Point-prevalence studies of post-flight barotitis in mixed adult and pediatric populations report incidence of 10–20%, with effective mitigation by autoinflation maneuvers (14). While the cochlear consequences are mediated indirectly, through pressure-driven round-window membrane displacement and rare perilymphatic fistula, the additive contribution of barotrauma to overall in-flight cochlear stress justifies its inclusion as an auxiliary environmental factor in this section.

### 2.4 Cosmic ionizing radiation dose climatology

Cosmic radiation dose rate at flight altitude is the joint product of the galactic cosmic-ray flux (modulated by solar activity through the heliocentric potential, HCP), the geomagnetic cut-off rigidity *R*_*c*_ (set by the geographic location of the route), and the residual atmospheric overburden (determined by cruise altitude). The U.S. FAA CARI-7 model (24) and the European NAIRAS framework (25, 26) are the standard physics-based tools for computing the effective dose rate *D*(*h, R*_*c*_, HCP). Building on these, we use in this work a screening-level closed-form exponential approximation to the CARI-7 altitude–dose relation under Solar Cycle 25 maximum conditions,


$$\begin{array}{l} D\left(h\right)=0.0424\exp\left(0.335h_{\text{km}}\right)\;\left[\mu \text{Sv}{\text{h}}^{-1}\right], \end{array}$$ (1)

with extensions for geomagnetic latitude correction *f*_lat_(*R*_*c*_) and solar modulation *f*_⊙_(HCP) that we adopt in Section 5. Equation 1 is a screening-level closed-form approximation to the CARI-7 altitude–dose relation and is benchmarked against the Korean-peninsula in-flight Liulin spectrometer measurements of Lee et al. (2.3 ± 0.17μSv h^−1^ at FL300) (27), shown as the anchor marker in Figure 1A; the predicted dose magnitudes are consistent with broader published aircrew dose climatologies and model intercomparisons (38, 39, 43, 44). Three representative cut-off rigidities are plotted: polar (*R*_*c*_ = 1.0 GV, characteristic of Svalbard and northern Canadian routes), mid-latitude Korea (*R*_*c*_ = 9.96 GV; reference for the present framework), and equatorial (*R*_*c*_ = 17.0 GV; Singapore/Nairobi class).

Two operationally relevant features emerge from this dose climatology. First, the exponential altitude dependence produces a 24.7 × range in dose rate between 10,000 ft and 40,000 ft, making cruise altitude the dominant determinant of cumulative dose. Second, polar routes systematically exceed 1 mSv yr^−1^ at FL300 even at moderate annual flight-hours, while equatorial routes can remain comfortably below the ICRP public reference level even at high-altitude cruise; the operational implication is that the *geographic* signature of the route is as important as its altitude profile for radiation-component prioritization.

#### 2.4.1 Quantification and validation of the radiation factor

The radiation exposure is quantified entirely from operational inputs derivable from flight-log metadata (cruise altitude, route-derived geomagnetic cut-off rigidity *R*_*c*_, solar-cycle phase (HCP) and segment flight-hours) through the closed-form CARI-7 approximation of Equations 1–5; no per-flight dose reconstruction or new measurement is required. The *dose* side is validated against published in-flight dosimetry: the Lee et al. Liulin measurement at FL300 (27) and broader model intercomparisons and compiled datasets (38, 39, 43, 44). The *cochlear-effect* side, whether this chronic low-dose exposure damages the cochlea, cannot yet be validated, because no audiometric or cochlear endpoint has been reported at μSv–mSv doses (Section 2.5); the appropriate data-collection routes are (i) a prospective aircrew audiometric cohort stratified by cumulative dose (Section 6) and (ii) accelerator-based galactic-cosmic-ray analog studies in animal models with auditory endpoints. Until such data exist the radiation axis is retained as exploratory, and the radiation-excluded (*w*_*R*_ = 0) scenario of Section 5.5 confirms that the conclusions do not depend on it.

### 2.5 Limited evidence linking cruise-altitude radiation to cochlear damage

Direct epidemiological evidence linking the cruise-altitude cosmic-radiation environment to human cochlear dysfunction is *not currently available*, and we say so explicitly. The aircrew cohorts of Pukkala et al., Hammer et al., and Langner et al. were designed to detect cancer endpoints rather than audiometric ones, and to our knowledge no aircrew study has reported pure-tone audiometry stratified by cumulative cosmic-radiation dose (19–22). What is available is a mechanism-level corpus drawn from clinical head-and-neck radiotherapy at much higher delivered cochlear doses. We are not aware of any study reporting auditory or cochlear endpoints from chronic low-dose (μSv–mSv) ionizing radiation; the accelerator-based galactic-cosmic-ray simulation studies (45, 46) report central-nervous-system rather than specifically cochlear outcomes. This low-dose cochlear data gap is one the present framework flags explicitly rather than fills.

#### 2.5.1 Dose-response evidence at therapeutic levels

The QUANTEC review of Bhandare et al. identified a clinically relevant transition near 35–45 Gy to the cochlea, above which sensorineural hearing loss prevalence rises steeply (28), and the systematic review of Theunissen et al. across 53 head-and-neck cohorts quantified the high-frequency-biased prevalence pattern reproduced in Figure 2B, with SNHL rising from 22% at 2 kHz to 67% at 8 kHz (29, 47, 48).

#### 2.5.2 Mechanism at the ribbon synapse

The 2022 study of Liu et al. (30) provided the first direct mechanistic demonstration that head-and-neck radiotherapy specifically disrupts cochlear ribbon synapses in C57BL/6J mice exposed to 10 and 20 Gy of ^60^Co radiation, with a high-frequency basal cochlear bias. The disruption was localized at the inner hair-cell–auditory-nerve interface and was quantified by anti-RIBEYE/CtBP2 immunolabeling. Complementary cellular work implicates reactive-oxygen-species pathways, mitogen-activated protein kinase signaling, and p53-mediated apoptosis as the intracellular cascade linking radiation-induced damage to cochlear hair-cell death and synapse loss (48, 49).

#### 2.5.3 Scope of the radiation hypothesis in the present work

Because the dose magnitude at cruise altitude is several orders of magnitude below the radiotherapy range that produces measurable clinical SNHL, we are explicit that the present paper does *not* claim cosmic radiation as an established aviation auditory hazard. We treat it as a *candidate* contributor whose frequency signature, if mechanistically active even at low chronic doses, would be high-frequency-biased and would co-localize with the classical noise-notch in the 3–6 kHz convergence band that the remainder of this paper formalizes. This positioning is consistent with the limited-evidence framing established in Section 1 and is preserved in the CCSI weighting choices reported in Section 5.

Together, Sections 2.1–2.5 establish that the aircrew cochlea is exposed to three temporally co-occurring stressors, each with documented or mechanistically plausible frequency-band signatures concentrated within 3–6 kHz. The quantitative integration of these three streams, and the resulting TSC-region prioritization across operational profiles, is the subject of Sections 4–6.

## 3 Threshold sound conditioning: current evidence

### 3.1 Mechanism of action: auditory pathway plasticity in adults

The mechanistic basis of threshold sound conditioning rests on a substantial body of work demonstrating that the adult mammalian auditory pathway retains an unexpectedly broad capacity for experience-dependent reorganization. Recanzone et al. first showed in adult owl monkeys that perceptual discrimination training induces measurable enlargement of the cortical representation of the trained frequency band in primary auditory cortex (33), establishing that tonotopic maps are not fixed at developmental closure. Twelve years later, Noreña and Eggermont demonstrated in a noise-trauma cat model that exposure to an enriched acoustic environment composed of tone-pips spanning the deprived frequency region during the post-trauma window both reduces permanent threshold shift and *prevents* the tonotopic-map reorganization that normally accompanies acoustic deprivation (31); the same group subsequently showed that this paradigm abolishes neural signatures of tinnitus (32). More recent reviews have framed these findings as defining a clinically actionable plasticity window in which targeted acoustic exposure can recruit residual cochlear function and stabilize central representations (34). The cochlear-synaptopathy literature has additionally established that the auditory nerve degenerates selectively after even temporary threshold shifts of seemingly modest magnitude, providing a second rationale for early intervention targeted at preserving the residual ribbon-synapse pool (50–53).

### 3.2 Clinical RCT evidence: the Kwak and Kwak 2020 trial

The clinical translation of these findings into a deployable intervention is captured in the Kwak & Kwak randomized controlled trial, conducted under Stanford University Medical Center IRB oversight and reported initially as an American Academy of Neurology abstract (36) and subsequently in extenso in *Laryngoscope Investigative Otolaryngology* (35). Ninety-six candidates were screened from a single tertiary-care neurology clinic; following application of audiometric and protocol-compliance criteria, 42 participants were randomized and 39 completed the study (23 TSC, 16 control). Inclusion required at least one pure-tone frequency threshold above 40 dB HL; exclusion criteria included three or more air-bone gaps exceeding 10–15 dB or three or more frequency regions with profound loss above 90 dB.

Pure-tone audiometry was performed at 0.25, 0.5, 1, 2, 3, 4, 6, 8, and 12 kHz before and after intervention. The TSC arm received a single tonal stimulus, individually selected at 3, 4 or 6 kHz to match the participant's poorest pure-tone threshold, delivered for 1 h per day via a behind-the-ear digital hearing aid for two to 3 weeks. The control arm received an identical hearing-aid platform without the TSC waveform. The primary endpoint was threshold amelioration defined under two dependent-variable formulations (DV1 and DV2) over the analyzed frequency set. Threshold amelioration was observed in 78% of the TSC arm vs. 44% in the control arm, with statistical separation by Scheffé *post-hoc* testing (*p* = 0.008091 in DV1, *p* = 0.000546 in DV2). A sub-group analysis showed that female participants exhibited a larger TSC benefit than male participants (77% vs. 47%, *p* = 0.025), while older and younger participants did not differ significantly. A published erratum clarified minor analytical-table corrections without altering the principal effect estimates (37). The study was graded Level of Evidence 1a, and remains the first prospective double-blind RCT to evaluate threshold-matched conditioning in sensorineural hearing loss.

### 3.3 Cochlear model and audiometric apparatus underpinning TSC

Translating the bench-side conditioning concept into a deployable clinical instrument required an audiometric and signal-processing pipeline capable of (i) resolving the patient's hearing threshold at sub-octave frequency resolution, (ii) selecting a conditioning band that matches the most affected cochlear region, and (iii) delivering the stimulus at strictly sub-threshold intensity without driving secondary acoustic trauma. Objective automated audiometry based on the wave-V and SN10 peaks of the auditory brainstem response (54) and a cochlear-model-based apparatus that divides the audible band at 1/*k*-octave resolution (55) provide the relevant technical basis; a complementary preprint provides a high-resolution stochastic model of tinnitus loudness derived from algorithmic pure-tone audiometry at 134 frequency bands (1/24-octave resolution) in 196 chronic tinnitus patients (56), the methodologically relevant precedent for the extended high-frequency audiometry recommended for the prospective aircrew cohort (Section 6). We cite this engineering background for completeness only; the CCSI framework is independent of any specific device implementation.

### 3.4 Why 3, 4, and 6 kHz?

The TSC region was empirically narrowed to the 3, 4, and 6 kHz options in the Kwak and Kwak 2020 protocol on the basis of the participants' audiometric profile: the worst observed pure-tone threshold among patients with sensorineural hearing loss consistently localized within this band, mirroring the dominant frequency band of noise-induced and presbycusis-related cochlear damage. The present paper recasts this empirical selection as a derivable consequence of multi-hazard cochlear physiology: under the convergence hypothesis advanced in Section 1 and developed quantitatively in Section 4, 3–6 kHz emerges naturally as the geometric overlap of noise, hypoxia and radiation vulnerability. Section 5 operationalises this overlap into a frequency-resolved score that selects the conditioning band from the operational flight profile rather than from a previously administered audiogram, motivating a prospective evaluation of TSC as a candidate preventive paradigm in aircrew.

## 4 Hypothesis: convergent 3–6 kHz vulnerability

### 4.1 Formal statement

The central hypothesis of the present paper can be stated as follows. Consider the auditory frequency axis discretized into eight standard audiometric bands f∈F={0.5,1,2,3,4,6,8,12} kHz, and consider three mechanism-distinct cochlear vulnerability kernels: *g*_*N*_(*f*) for cabin acoustic noise, *g*_*H*_(*f*) for hypobaric hypoxia, and *g*_*R*_(*f*) for ionizing radiation. Each kernel is parameterized as a Gaussian peak in log-octave frequency space,


$$\begin{array}{l} g_{X}\left(f\right)=\exp\left[-\frac{1}{2}(\frac{\log_{2}\left(f/\mu_{X}\right)}{\sigma_{X}}{)}^{2}\right],\;X\in \left\{N,H,R\right\}, \end{array}$$ (2)

with kernel parameters (μ_*X*_, σ_*X*_) calibrated to the mechanistic-evidence corpus reviewed in Section 2: (μ_*N*_, σ_*N*_) = (4kHz, 0.8oct) for the classical noise-notch (1, 2), (μ_*H*_, σ_*H*_) = (3kHz, 1.0oct) for the hypobaric-hypoxia threshold-shift band (10–12), and (μ_*R*_, σ_*R*_) = (6kHz, 0.9oct) for the high-frequency-biased ribbon-synapse damage observed in head-and-neck radiotherapy (28–30).

Define the per-frequency convergence index


$$\begin{array}{l} g_{\text{conv}}\left(f\right)=[g_{N}\left(f\right)g_{H}\left(f\right)g_{R}\left(f\right){]}^{1/3}, \end{array}$$ (3)

i.e. the geometric mean of the three kernels.

Convergence hypothesis (*H*_1_): The geometric-mean convergence index *g*_conv_(*f*) attains its maximum within the Kwak & Kwak clinical TSC region $F_{\text{TSC}}=\left\{3,4,6\right\}$kHz, i.e.


$$\begin{array}{l} \arg\underset{f\in F}{\max}g_{\text{conv}}\left(f\right)\in F_{\text{TSC}}. \end{array}$$ (4)

Null hypothesis (*H*_0_): The argmax of *g*_conv_(*f*) falls outside the clinical TSC region, indicating that the empirical Kwak & Kwak frequency selection is not derivable from multi-hazard cochlear physiology and must be motivated solely by *post-hoc* audiometric observation.

### 4.2 Numerical evaluation

Evaluating Equation 3 on a logarithmically spaced fine grid (300 points over 0.5–12 kHz) yields a smooth curve peaking at f^*^ = 4.23 kHz with $g_{\text{conv}}\left(f^{*}\right)=0.91$ on the normalized scale; the analytical solution $\log_{2}f^{*}=(\underset{X}{\sum }\sigma_{X}^{-2}\log_{2}\mu_{X})/(\underset{X}{\sum }\sigma_{X}^{-2})$ yields the same value to three decimal places. The full-width at half-maximum spans 2.06–8.72 kHz, fully containing the clinical TSC region; the curve and the underlying three kernels are plotted in Figure 2C. Restricted to the discrete audiometric grid F, the argmax lies at 4 kHz, which is the central element of the clinical TSC set $F_{\text{TSC}}$. The per-band values of *g*_conv_ on the discrete grid are {0.002, 0.059, 0.434, 0.778, 0.906, 0.774, 0.533, 0.217} for *f* ∈ {0.5, 1, 2, 3, 4, 6, 8, 12} kHz, respectively. This calculation is not a statistical test of *H*_1_ but a formal consistency check showing that the three independently motivated kernel priors converge inside the clinically used TSC frequency set; the substantive content of *H*_1_ is discussed in Section 4.3.

### 4.3 Why the hypothesis is non-trivial

A reasonable objection is that the kernels in Equation 2 are themselves *set* from the same audiometric literature that defines $F_{\text{TSC}}$, making *H*_1_ tautological. We acknowledge this and clarify what the hypothesis does and does not claim.

- The kernel peak frequencies μ_*N*_, μ_*H*_, μ_*R*_ are *not* derived from the Kwak and Kwak clinical protocol; they are independently calibrated to noise-induced audiometric prevalence [μ_*N*_ = 4 kHz from Falcão *n* = 3, 130 (1)], hypobaric-hypoxia threshold-shift maps [μ_*H*_ = 3 kHz from ADEMED 2011 and Singh 2021 (10, 11)], and ribbon-synapse damage maps from radiotherapy [μ_*R*_ = 6 kHz from Liu 2022 (30) and the high-frequency-biased prevalence pattern of Theunissen (29)]. The convergence onto 3–6 kHz is therefore a *consequence* of three independent mechanistic literatures rather than a circular reading of the clinical protocol.
- The non-trivial claim is the *geometric overlap* of three kernels with different peak frequencies (3, 4, and 6 kHz) within a finite band that coincides with the empirical clinical selection set. A natural counter-hypothesis would be a noise-only or hypoxia-only single-kernel argmax that falls outside 3–6 kHz, which would render $F_{\text{TSC}}$ a clinical-only construct. *H*_1_ rules out this alternative.
- A further non-trivial claim, developed quantitatively in Section 5, is that the kernel-weighted multi-hazard score *shifts* the argmax in a profile-specific way across the clinical TSC set. The framework therefore generates a profile-level prediction (3 vs. 4 vs. 6 kHz) that is testable in any prospective audiometric cohort.

#### 4.3.1 Monte-Carlo test of non-triviality

To quantify the tautology objection directly we performed a Monte-Carlo randomization of the kernel parameters (*N* = 2 × 10^5^ draws) and recorded how often the discrete-grid argmax of *g*_conv_ falls inside $F_{\text{TSC}}=\left\{3,4,6\right\}$ kHz. Under *mechanistic* uncertainty, peaks drawn uniformly within the literature-reported ranges (μ_*N*_ ∈ [3.5, 4.5], μ_*H*_ ∈ [2.5, 3.5], μ_*R*_ ∈ [5, 7] kHz, σ_*X*_ ∈ [0.7, 1.1] oct), the argmax lies inside $F_{\text{TSC}}$ in 100.0% of draws and lands specifically at 4 kHz in 99.2%, confirming that the convergence is robust to the exact kernel specification rather than an artifact of a single parameter choice. Under an *unconstrained* null, all three peaks drawn log-uniformly over the 1–8 kHz audiometric range, the argmax falls inside $F_{\text{TSC}}$ in only 64.6% of draws and at 4 kHz in only 23.1%. The contrast is informative and we report it without overstatement: landing *somewhere* in 3–6 kHz is only moderately non-trivial (a 64.6% chance baseline reflects the central position of the TSC bands on the audiometric grid), but the *specific* concentration at 4 kHz under mechanistically constrained kernels (99.2% vs. a 23.1% chance baseline, a 4.3 × enrichment) is strongly non-trivial and is the quantitative core of the convergence claim. The randomization routine and results are released as null_test.py and null_test_results.json in the deposit.

### 4.4 Relationship between *g*_conv_(*f*) and the operational CCSI

The convergence index *g*_conv_(*f*) in Equation 3 and the Cumulative Cochlear Stress Index CCSI(*f*) that we develop quantitatively in Section 5 are formally distinct mathematical objects with complementary roles. The convergence index is the geometric mean of the three vulnerability kernels and is *dose-independent*; it captures the intrinsic frequency-domain overlap among the three mechanism-distinct pathways and is the natural object on which hypothesis *H*_1_ is stated, as it tests whether the three kernels co-localize on a clinically defined frequency set regardless of how much each pathway actually contributes in a given operational scenario. The CCSI of Equation 9, in contrast, is an *operational, dose-weighted* arithmetic sum that multiplies each kernel by its corresponding per-segment hazard dose (*D*_*R*_, *D*_*H*_, *D*_*N*_) and a mixing weight *w*_*X*_; its argmax is profile-specific and shifts across the clinical $F_{\text{TSC}}$ set as the relative dose magnitudes change between aircrew operational profiles. We use *g*_conv_ to test the convergence hypothesis *H*_1_ and CCSI to derive the per-profile TSC recommendation. The two objects coincide in the limiting case where the three normalized doses are equal and the weights are uniform; outside this limit they encode different information.

### 4.5 Calibration assumptions and limitations of the kernels

The kernel parameters (μ_*X*_, σ_*X*_) in Equation 2 are chosen to summarize the dominant frequency emphasis of each pathway as reported in the cited literature, but they should be regarded as informed *prior* choices rather than as quantitatively fitted parameters. The peak μ_*N*_ = 4 kHz is well-anchored in the aircrew NIHL literature (1, 2). The peak μ_*H*_ = 3 kHz reflects the lower-mid-frequency emphasis of the hypobaric-hypoxia threshold-shift band reported by ADEMED 2011 (10) and the persistent 2–4 kHz elevation reported by Singh et al. (11); the choice of a single μ_*H*_ = 3 kHz is a working summary of a band-spread effect rather than a sharp localization. We further note that ADEMED 2011 acclimatized volunteers at 5,300 m, whereas the regulatory cabin-altitude cap corresponds to ≈2,440 m; the alveolar hypoxia index at the cabin cap (*H*≈0.45) is substantially milder than at the ADEMED altitude (*H*≈0.74 at 5,300 m, an ≈40% reduction at the regulatory cap), so μ_*H*_ and the hypoxia weighting should be read as a transfer of the threshold-shift *frequency emphasis* rather than a dose-matched extrapolation. This altitude gap is carried as an explicit uncertainty and is spanned by the μ_*H*_ ∈ [2.5, 3.5] kHz randomization in the Monte-Carlo robustness test. The peak μ_*R*_ = 6 kHz reflects the high-frequency-biased ribbon-synapse damage demonstrated by Liu et al. (30) and the monotonic high-frequency rise in radiotherapy-induced SNHL prevalence reported by Theunissen et al. (29); the value μ_*R*_ = 6 kHz positions the radiation kernel toward the high-frequency end without collapsing onto the 8–12 kHz range where audiometric coverage in the eligible aircrew is sparse. The widths σ_*X*_ (0.8–1.0 octave) are chosen so that all three kernels retain non-negligible support across 2–8 kHz, consistent with the broad audiometric signatures reported in each source literature; they are not independently fitted, and the impact of alternative widths is absorbed into the sensitivity analysis of Section 5.5.

## 5 Computational framework: CCSI

To mitigate the commercial conflict of interest declared at the end of the manuscript, the framework was developed under explicit independence safeguards. (i) The synthetic-dataset generation, the radiation and hypoxia dosimetry, the CCSI implementation, the machine-learning identifiability check, and the sensitivity and Monte-Carlo analyses were carried out by the non-conflicted, Chemical, Biological, Radiological and Nuclear (CBRN) Defense Research Institute-affiliated authors, who retained sole custody of the analysis code and input data. (ii) The authors affiliated with the commercial TSC developers (Sound Vaccine/AudioCardio) contributed clinical and audiometric domain expertise, the TSC clinical protocol (Section 3) and the literature anchoring of the cochlear-vulnerability kernels, but did not implement or run the dosimetric, machine-learning, or statistical analyses; moreover, the convergence result is robust to the kernel specification (Monte-Carlo test, Supplementary material), bounding the influence of any domain-expert kernel choice on the conclusions. (iii) The quantitative results, the conclusions, and the profile-specific clinical recommendation were drafted and approved by the non-conflicted authors. (iv) No commercial product data, device, or funding was used; the complete code and data are released openly (see the Data Availability Statement) so that every reported number can be reproduced independently of any author.

### 5.1 Operational inputs and physics dose proxies

For an aircrew profile indexed by cruise altitude *h* (km), flight-hours per segment *t* (h), geomagnetic cut-off rigidity *R*_*c*_ (GV), heliocentric potential HCP (MV) and cabin A-weighted equivalent level *L*_*Aeq*_ (dBA), we define three physics-based per-segment hazard “doses”.

#### 5.1.1 Radiation dose

We use a screening-level empirical approximation to the CARI-7 dose rates (24), parameterized as the closed-form altitude–dose relation of Equation 1 multiplied by geomagnetic-latitude and solar-modulation factors:


$$\begin{array}{l} D_{R}\left(h,R_{c},\text{HCP};t\right)=0.0424\exp\left(0.335h_{\text{km}}\right)f_{\text{lat}}\left(R_{c}\right)f_{⊙}\left(\text{HCP}\right)\cdot t, \end{array}$$ (5)

with $f_{\text{lat}}\left(R_{c}\right)=\left(1+aR_{c,\text{ref}}^{n}\right)/\left(1+aR_{c}^{n}\right)$, *a* = 0.0994, *n* = 1.289, *R*_*c*, ref_ = 9.96 GV (Korean Peninsula reference), and a solar-modulation factor *f*_⊙_(HCP) = 1+0.13(HCP_max_−HCP)/(HCP_max_−HCP_min_) with HCP_max_ = 1141 MV and HCP_min_ = 270 MV. The per-segment dose *D*_*R*_ is normalized by 1000μSv so that one normalized unit corresponds to a typical aircrew yearly exposure of ~1 mSv at FL300.

#### 5.1.2 Hypoxia dose

The cabin altitude *h*_cab_ = min(*h, h*_cap_) with *h*_cap_ = 2.44 km is fed into a simplified alveolar gas equation that returns the estimated alveolar oxygen tension *P*_*A*_*O*_2_,


$$\begin{array}{l} P_{A}O_{2}\left(h_{\text{cab}}\right)=F_{i}O_{2}(P_{\text{atm}}\left(h_{\text{cab}}\right)-47)-P_{a}CO_{2}/\text{RQ}, \end{array}$$ (6)

with $P_{\text{atm}}\left(h\right)=760e^{-h/7.4}$ mmHg, *F*_*i*_*O*_2_ = 0.2095, *P*_*a*_*CO*_2_ = 40 mmHg, RQ = 0.8; arterial *P*_*a*_*O*_2_ is somewhat lower due to the A–a gradient and is not separately modeled here. The alveolar hypoxia index *H*(*h*) = 1−*P*_*A*_*O*_2_(*h*_cab_)/*P*_*A*_*O*_2_(0) is then time-integrated to yield the per-segment hypoxia dose


$$\begin{array}{l} D_{H}\left(h;t\right)=H\left(h\right)\cdot t/\left(8\cdot H_{\text{cap}}\right),\;H_{\text{cap}}\approx 0.45, \end{array}$$ (7)

normalized so that one unit corresponds to an 8-h flight at the cap-equivalent hypoxia index *H*_cap_ = 1 − 55/99 ≈ 0.45 computed from Equation 6 at the regulatory cap altitude.

#### 5.1.3 Noise dose

The cabin acoustic environment is summarized by a single *L*_*Aeq*_, above a 65 dBA reference floor, and integrated over the flight segment,


$$\begin{array}{l} D_{N}\left(L_{Aeq};t\right)=\max\left(0,L_{Aeq}-65\right)/30\cdot t/8, \end{array}$$ (8)

normalized so that one unit corresponds to an 8-h segment at *L*_*Aeq*_ = 95 dBA.

### 5.2 Cumulative cochlear stress index

The frequency-resolved Cumulative Cochlear Stress Index combines the three hazard doses with the vulnerability kernels of Equation 2 via fixed mixing weights **w** = (*w*_*R*_, *w*_*H*_, *w*_*N*_):


$$\begin{array}{l} \text{CCSI}\left(f\right)=w_{R}D_{R}g_{R}\left(f\right)+w_{H}D_{H}g_{H}\left(f\right)\\ \;+w_{N}D_{N}g_{N}\left(f\right) \end{array}$$ (9)

where the inputs (*h, t, R*_*c*_, HCP, *L*_*Aeq*_) enter through the hazard doses *D*_*R*_, *D*_*H*_, *D*_*N*_ via Equations 5–8, and the kernels *g*_*X*_(*f*) are given by Equation 2. with *w*_*R*_, *w*_*H*_, *w*_*N*_≥0 and *w*_*R*_ + *w*_*H*_ + *w*_*N*_ = 1.

The default weighting reflects the relative *prior confidence* the literature supports for each hazard pathway and is intentionally distinct from the per-flight dose magnitudes that the framework generates. The noise pathway is the most empirically supported (decades of audiometric surveys in aircrew), yielding *w*_*N*_ = 0.45; the radiation pathway has strong mechanism evidence at therapeutic doses (Gy-scale fractionated radiotherapy) but no direct epidemiological link at the μSv–mSv scale of cruise exposure, so *w*_*R*_ = 0.35 should be read as an *illustrative prior placeholder, not as an evidence-derived biological weight*; sensitivity to this choice is examined in Section 5.5. To preserve independence from the declared commercial conflict of interest, all mixing weights were assigned by the non-conflicted authors (K. Kang, E. Choi, C.-M. Yang, J. Yoo, S. Kim and D.-H. Lee), and the radiation-excluded scenario (*w*_*R*_ = 0) of Section 5.5 confirms that the conclusions hold with the radiation weight removed entirely. The hypoxia pathway has the mechanistic support of ADEMED 2011 but is partially clipped by the regulatory cabin-altitude cap, yielding *w*_*H*_ = 0.20. The robustness analyses below confirm that the convergence-into-TSC claim does not depend on this weight assignment.

#### 5.2.1 Resulting magnitude breakdown

For a representative mid-latitude long-haul profile (FL360, *R*_*c*_ = 9.96 GV, HCP = 1141 MV, *t* = 8 h, *L*_*Aeq*_ = 78 dBA), the normalized per-segment doses are *D*_*R*_≈0.014, *D*_*H*_≈1.00 and *D*_*N*_≈0.43 under the cap-anchored hypoxia normalization of Equation 7. The 4 kHz CCSI contribution breakdown is *D*_*N*_*N*_*N*_≈0.195 (noise, 51% of total), *D*_*H*_*H*_*H*_≈0.183 (hypoxia, 48%) and *D*_*R*_*R*_*R*_≈0.004 (radiation, ~1%). Noise and hypoxia therefore contribute comparably at 4 kHz under default weights, with radiation entering as a small mechanism-bearing term consistent with the limited evidence at aviation doses. For the short-haul military-fighter/rotary profile (*t* = 1.5 h, *L*_*Aeq*_ = 92 dBA), the same accounting yields a noise-dominated breakdown in which the time-integrated hypoxia advantage shrinks and the noise contribution overtakes the hypoxia contribution at 4 kHz. The framework is therefore not exclusively noise-driven: under long-haul profiles noise and hypoxia are comparable, while under short-haul high-noise profiles the noise pathway dominates. In both regimes the argmax of CCSI within the clinical TSC set $F_{\text{TSC}}$ remains controlled by the geometric overlap of the three kernels rather than by any single pathway, which is the central operational consequence of the convergence hypothesis *H*_1_.

Sensitivity analyses across alternative weight choices reproduce the 3–6 kHz argmax robustly (Section 5.5); the framework's qualitative output therefore does not hinge on the exact weight values.

### 5.3 Synthetic dataset

**Figure 3:**
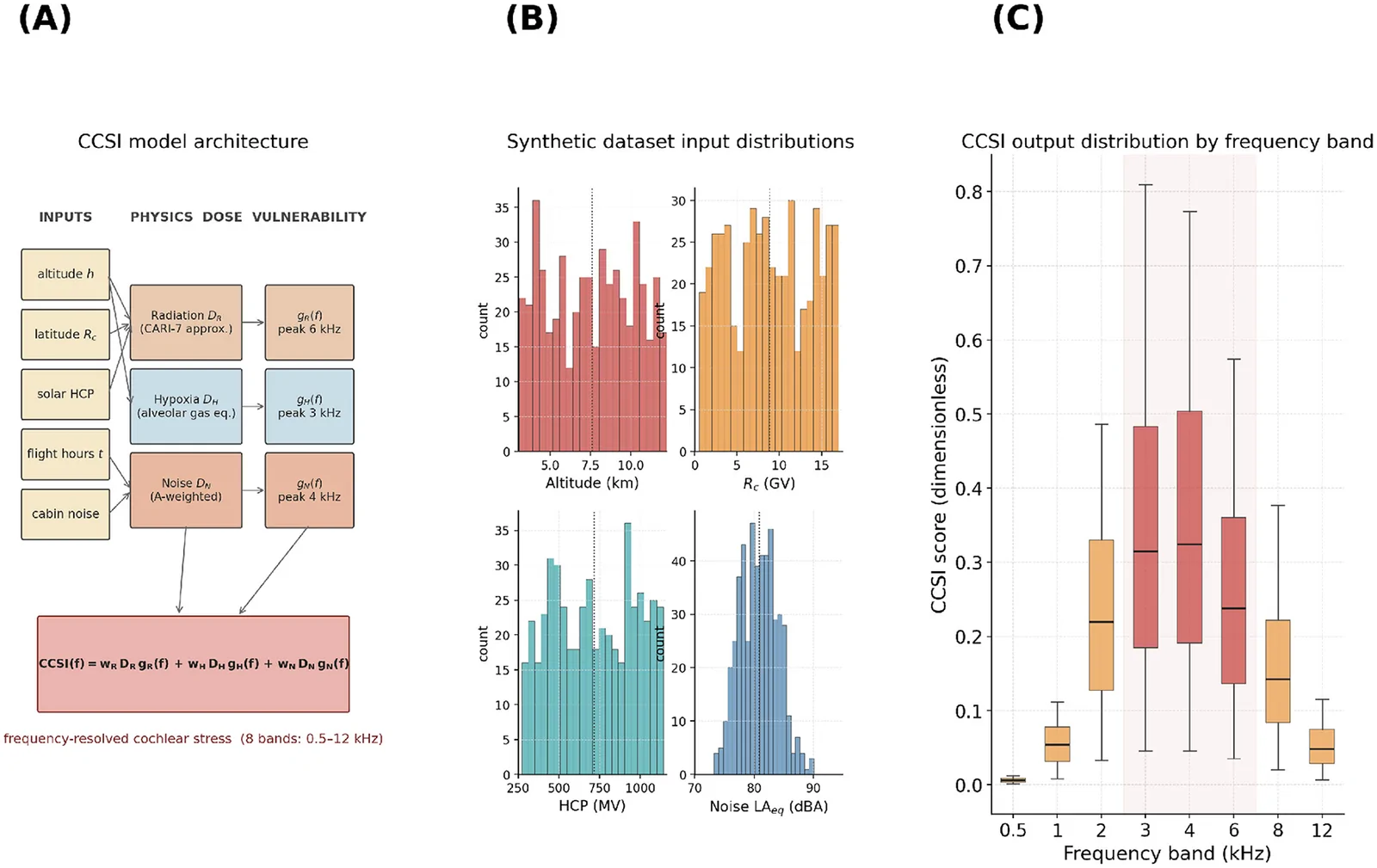
CCSI model architecture and synthetic dataset. **(A)** Schematic of the input–proxy–kernel–output flow. **(B)** Marginal distributions of the four primary input features over *n* = 500 uniformly sampled aircrew profiles. **(C)** Per-frequency-band distribution of CCSI labels, with the 3–6 kHz Kwak TSC region shaded.

To enable per-feature, per-band interpretation of the framework and to visualize the operational envelope (Figures 1B, 3B, C), we constructed a synthetic dataset of *n* = 500 aircrew flight profiles by uniform sampling over operational ranges of the five inputs: *h* ∈ [3.0, 12.2] km, *t* ∈ [1, 12] h, *R*_*c*_ ∈ [0.5, 17] GV, HCP ∈ [270, 1141] MV, and a mild altitude-dependent cabin-noise term anchored to GAO 2018 ($L_{Aeq}=74+0.9h_{\text{km}}+N\left(0,2.0\right)$, clipped to [60, 100] dBA). Each sample is labeled by Equation 9 with σ = 5% multiplicative Gaussian noise applied to the per-band CCSI labels to represent intra-population biological variability and CARI-7-grid interpolation uncertainty. The dataset and the generation routine are released through the Zenodo deposit referenced in the *Data Availability Statement* section. A machine-learning identifiability check trained on this dataset is reported in Supplementary material; it is an internal model-recovery analysis rather than part of the substantive argument of the main text.

### 5.4 Model architecture summary

Figure 3 summarizes the framework. Panel A is the schematic of the five operational inputs, the three physics dose proxies (Equations 5–8), the three vulnerability kernels (Equation 2), and the final weighted sum (Equation 9). Panel B reports the marginal distributions of the four primary input features over the *n* = 500 synthetic sample, confirming uniform coverage of the operational envelope. Panel C shows the per-frequency-band distribution of CCSI labels, with a clear maximum in the 3–6 kHz band; the inter-quartile spread within each band reflects the variance contribution of *h*, *t* and *L*_*Aeq*_.

### 5.5 Sensitivity to mixing weights

The mixing weights **w** in Equation 9 are intentionally specified as fixed prior weights rather than as free fitted parameters. To characterize the robustness of the profile-level prioritization against this prior choice, we evaluated Equation 9 for the four representative operational profiles of Table 1 under six mixing-weight scenarios that span the physiologically defensible region of the weight simplex. For each profile × weight combination we computed the *global* argmax of CCSI over the full discrete audiometric grid F={0.5,1,2,3,4,6,8,12}kHz, not restricted to $F_{\text{TSC}}=\left\{3,4,6\right\}$ kHz, so that a non-trivial robustness check is performed: a result $\arg\underset{f\in F}{\max}\text{CCSI}\left(f\right)\in F_{\text{TSC}}$ would not be true by construction. The result is summarized in Table 2.

**Table 1:** Representative aircrew operational profiles used for CCSI prioritization.

Profile	*h* (km)	*R*_*c*_ (GV)	HCP (MV)	*t* (h)	LA_*eq*_ (dBA)
Polar long-haul	11.0	1.00	600	10.0	78
Mid-latitude long-haul	11.0	9.96	1141	8.0	78
Domestic short-haul	8.5	9.96	1141	1.5	80
Military fighter/rotary	6.0	9.96	1141	1.5	92

Columns: cruise altitude *h*, geomagnetic cut-off rigidity *R*_*c*_, heliocentric potential HCP, per-segment flight hours *t*, cabin LA_*eq*_.

**Table 2:** Sensitivity of the global CCSI argmax (kHz; $\arg\underset{f\in F}{\max}\text{CCSI}\left(f\right)$ over the full discrete audiometric grid F={0.5,1,2,3,4,6,8,12}kHz, not restricted to $F_{\text{TSC}}$) to the mixing weights **w** = (*w*_*N*_, *w*_*H*_, *w*_*R*_) across the four aircrew profiles of Table 1.

Weight scenario	(*w*_*N*_, *w*_*H*_, *w*_*R*_)	Polar long-haul	Mid-lat long-haul	Domestic short-haul	Military fighter	Pattern summary
Default	(0.45, 0.20, 0.35)	4	4	4	4	4/4/4/4
Balanced	(0.40, 0.30, 0.30)	4	3	4	4	4/3/4/4
Noise-extreme	(0.50, 0.10, 0.40)	4	4	4	4	4/4/4/4
Radiation-extreme	(0.30, 0.20, 0.50)	4	4	4	4	4/4/4/4
Hypoxia-extreme	(0.30, 0.50, 0.20)	3	3	3	3	3/3/3/3
Radiation-excluded	(0.69, 0.31, 0.00)	4	4	4	4	4/4/4/4

All 24 cells lie within $F_{\text{TSC}}=\left\{3,4,6\right\}$ kHz, providing a non-tautological robustness check. Per-cell CCSI values for all eight bands are released in sensitivity_table.csv.

Two observations follow. First, in all 24 profile × weight combinations the global CCSI argmax over the full audiometric grid F lies inside $F_{\text{TSC}}=\left\{3,4,6\right\}$ kHz; the convergence-into-TSC-region claim is therefore robust to the weight prior in a non-tautological sense. Second, the precise band selection within $F_{\text{TSC}}$ is weight-sensitive at the extremes: under default, noise-extreme and radiation-extreme priors the argmax falls on 4 kHz across all four profiles, while under a hypoxia-extreme prior it shifts to 3 kHz across all four. The balanced prior produces a mixed 4/3/4/4 pattern, with the mid-latitude long-haul profile, where time-integrated hypoxia and noise are most evenly matched, being the only one that shifts. No weight scenario in the simplex examined here places the global argmax outside $F_{\text{TSC}}$. Critically, a *radiation-excluded* scenario (*w*_*R*_ = 0, with the noise and hypoxia weights renormalized to 0.69:0.31) reproduces the 4 kHz argmax for all four profiles, demonstrating that the convergence and the resulting TSC recommendation do *not* depend on the radiation axis, the most heavily caveated and commercially sensitive component, at all.

The pattern in Table 2 clarifies what the framework does and does not assert. The convergence hypothesis *H*_1_ (Section 4) is robust across the whole weight simplex examined here, in the non-tautological sense that the *global* argmax, unconstrained to the TSC set, lands inside $F_{\text{TSC}}$ in every cell. The clinical recommendation of the precise band within $F_{\text{TSC}}$, however, is sensitive to the weighting prior; under default weights the framework selects 4 kHz for all four representative profiles, while a hypoxia-dominant prior shifts the selection uniformly to 3 kHz. For operational deployment we therefore recommend reporting the recommended frequency under the default weights together with the extreme-prior bracket as an uncertainty range.

### 5.6 Aircrew profile prioritization

**Figure 4:**
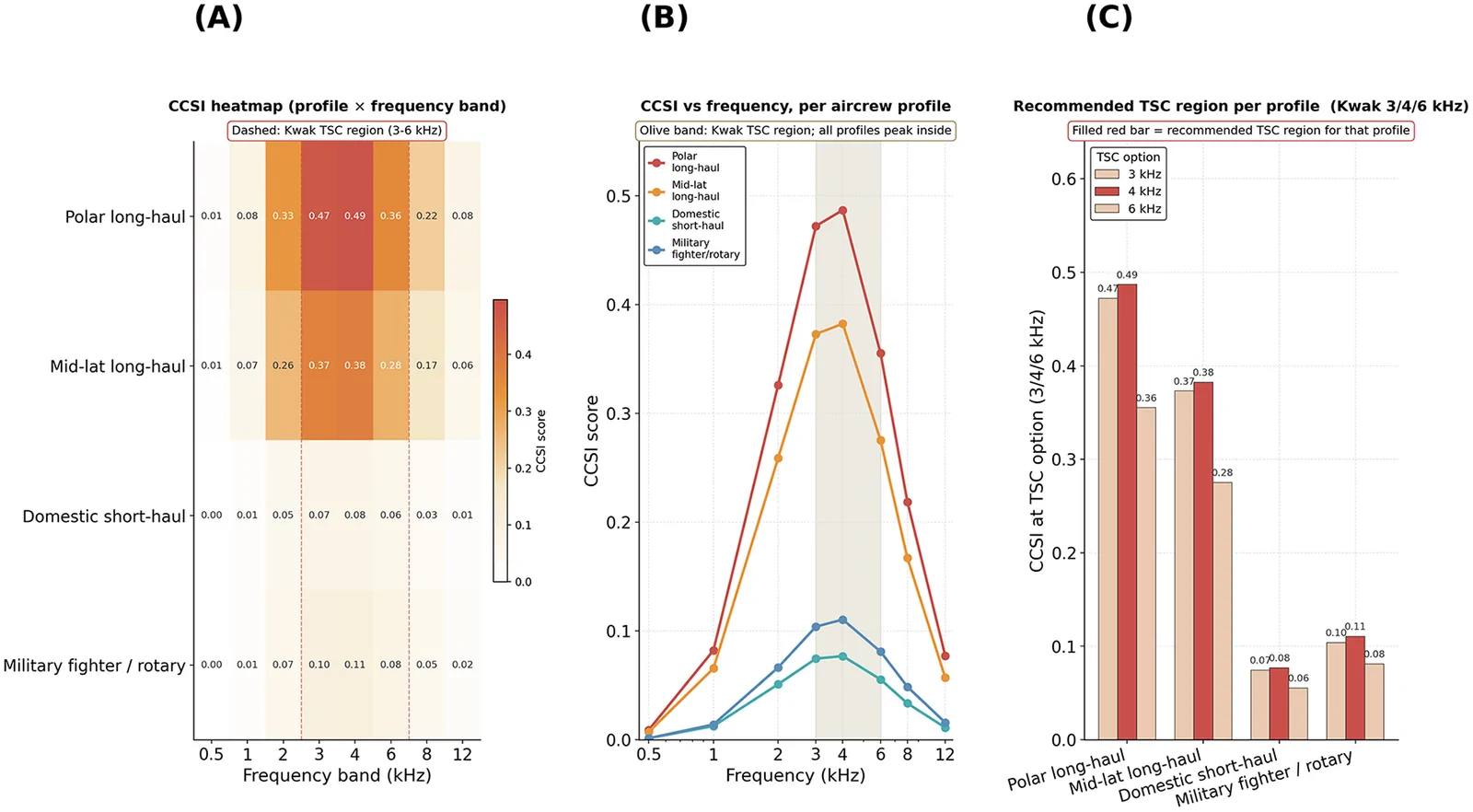
TSC region prioritization across representative aircrew operational profiles. **(A)** CCSI heatmap of four profiles (polar long-haul, mid-latitude long-haul, domestic short-haul, military fighter/rotary) by frequency band; dashed verticals mark the Kwak TSC region. **(B)** Per-profile CCSI–frequency curves overlaid. **(C)** Recommended TSC region per profile within the clinical 3/4/6 kHz set; the filled red bar indicates the selected target frequency, and the boxed label above each profile reports the recommendation.

Application of Equation 9 to four representative operational profiles defined in Table 1 (and instantiated in the released code) produces the CCSI heatmap of Figure 4A and the per-profile CCSI–frequency overlay of Figure 4B; the recommended TSC region per profile, restricted to the clinical set $F_{\text{TSC}}=\left\{3,4,6\right\}$kHz, is reported in Figure 4C. Under the default literature-prior weighting all four profiles place the within-$F_{\text{TSC}}$ argmax at 4 kHz, reflecting the geometric overlap of the three vulnerability kernels at the canonical noise-notch frequency. The 3 kHz band is the closest neighbor: at 4 kHz it lags by under 1–3% of the CCSI at the argmax across these profiles (see sensitivity_table.csv), so under a hypoxia-dominant prior the selection shifts uniformly to 3 kHz (Table 2). The 4 kHz default selection is consistent with the dominant 4 kHz noise-notch pattern reported in both civilian and military aircrew cohorts (1, 2). Profile-specific clinical implications and the rationale for reporting the recommendation as a default-plus-bracket are developed in Section 6.

## 6 Implications and research agenda

The CCSI framework developed in Sections 4–5 provides three distinct categories of operational implication, each of which suggests a concrete next step.

### 6.1 Translational implication: TSC as a candidate preventive intervention in aviation medicine

The Kwak and Kwak 2020 clinical protocol selects the TSC target frequency from the patient's worst-threshold band within 3, 4, or 6 kHz (35). The CCSI framework reproduces this clinical decision rule *from the operational flight profile alone*, without requiring a pre-existing audiogram. Across the four representative profiles in Figure 4C, the CCSI argmax under default weights falls on 4 kHz for all four representative profiles, in agreement with the dominant 4 kHz noise-notch pattern reported in both civilian and military aircrew cohorts (1, 2). Under a hypoxia-dominant weighting the recommendation shifts uniformly to 3 kHz (Table 2), and the 3 kHz band remains within 1–3% of the 4 kHz argmax across all four default-weight profiles. The framework therefore identifies 4 kHz as the default target and 3 kHz as a defensible alternative when hypoxia exposure is judged dominant, suggesting that the CCSI argmax could serve as a profile-specific prior in a prospective evaluation of TSC.

Two operational extensions follow. First, for long-haul commercial aircrew already enrolled in occupational audiometric surveillance, the CCSI argmax provides a flight-profile-derived recommendation that can be cross-checked against the audiometric finding when surveillance begins to detect threshold drift; the CCSI prior thus functions as a candidate *pre-emptive* target rather than a reactive one, conditional on prospective audiometric validation. Second, for short-haul or rotary-wing crews in whom long-haul-style audiometric surveillance is operationally impractical, the CCSI-recommended frequency provides a hypothesis-generating target on the basis of the cumulative LA_*eq*_ in the dominant flight envelope, without requiring a per-individual audiogram.

Practically, the CCSI is intended to *complement*, not replace, existing hearing-conservation practice. Within current occupational programs, periodic pure-tone and extended high-frequency audiometry, noise dosimetry, and fitness-to-fly review, a validated CCSI could (i) triage surveillance intensity, flagging the routes and crews (e.g. polar long-haul, high-*L*_*Aeq*_ rotary) whose profiles predict the steepest 3–6 kHz risk; (ii) supply a pre-audiometric prognostic prior that is cross-checked against, and updated by, each surveillance audiogram, sharpening early detection of threshold drift; and (iii) guide frequency-targeted countermeasures, scheduling or hearing-protection adjustments and, where indicated, a profile-matched TSC target, ahead of measurable loss. In this role the framework adds a frequency-resolved, exposure-driven dimension to programs at present triggered reactively by audiometric change.

### 6.2 Epidemiological implication: a prospective aircrew audiometric cohort

The most direct empirical test of the convergence hypothesis would be a prospective audiometric cohort that recruits aircrew across the four operational profiles characterized in Figure 4 and follows their pure-tone thresholds longitudinally over a period long enough to resolve cumulative exposure. Two design features are critical for such a cohort.

First, the audiometric protocol must include extended high-frequency audiometry (9–18 kHz), because high-frequency damage from cumulative exposure consistently precedes conventional pure-tone loss (2, 7, 8). High-resolution automated audiometry at sub-octave resolution, as described in the companion preprint (56), provides a methodologically suitable testing approach; we report this engineering precedent without endorsing any specific commercial platform.

Second, the cohort must record the four CCSI input features (cumulative flight-hours, route-distribution-derived *R*_*c*_, solar-cycle phase via HCP, and route-typical LA_*eq*_). The screening-level empirical approximation of Equations 1–5 reduces the radiation component to closed-form inputs that can be derived from flight-log metadata alone, avoiding the per-flight dose reconstruction otherwise required.

Third, the cohort should be powered for the primary frequency-shift endpoint. As an illustrative benchmark, detecting a between-arm difference equivalent to the Kwak and Kwak 2020 amelioration contrast (78% vs. 44%) at α = 0.05 (two-sided) with 80% power requires ≈16 evaluable participants per arm by a two-proportion test; allowing for ~20% attrition and stratification across the four operational profiles points to a target enrolment of ~150 aircrew. A formal power analysis indexed to the profile-specific CCSI argmax is the natural next design step.

Cross-validation against the existing Pukkala Nordic cohorts (19–21), which record cumulative radiation dose but not audiometric outcomes, would require audiometric retro-fitting, which is not generally feasible. A new prospective cohort with both inputs and outcomes is the cleaner study design.

### 6.3 Regulatory implication: dose-aware screening thresholds

The CARI-7-derived screening approximation of Equations 1–5 implies that polar long-haul operations systematically exceed the ICRP public reference level of 1 mSv yr^−1^. The companion CCSI framework presented here adds a frequency-resolved auditory dimension to this observation and supports the case for systematic cochlear monitoring on the subset of routes that combine *R*_*c*_ < 4.5 GV (i.e. northern Europe and polar) with sustained cruise altitude above ~ 11 km. Importantly, exceedance of the radiation reference level is not by itself evidence of clinically relevant cochlear harm; rather, it is a defensible trigger for enhanced monitoring of the very pathway (3–6 kHz cochlear vulnerability) that the convergence hypothesis predicts.

### 6.4 Methodological extensions

Three modeling refinements are within scope of the present framework. First, the hypoxia component of CCSI is presently parameterized through the cabin-altitude regulatory cap, which makes the hypoxia contribution near-constant across cruise altitudes within the commercial envelope and limits the discriminative power of the hypoxia feature in the ML decomposition (Supplementary Figure S1C). A prospective extension that records actual cabin-altitude variation across operators and aircraft types (which is known to span 6,000–8,000 ft equivalent) would restore variance to this feature and clarify its contribution.

Second, the cabin-noise input is presently a single LA_*eq*_ value, which collapses spectral content into a single number. An octave-band-resolved noise input, fed through a band-matched vulnerability kernel rather than a single 4 kHz Gaussian, would sharpen the noise-component frequency signature and likely separate the wide-body cabin (engine-broadband) from the fighter/rotary cockpit (gear-discrete and pulsed) operational patterns more cleanly.

Third, the radiation component is presently anchored to the CARI-7-derived screening approximation of Equations 1–5 and assumes that the cochlear damage function follows a Gaussian band centered at 6 kHz, inferred from the high-dose radiotherapy literature (28–30). Validation of this extrapolation at low chronic doses is, as noted in Section 2.5, not currently available, and a calibration study using accelerator-based GCR analog (45, 46) in animal models would be a natural follow-up. A further extension is to couple the CCSI score to biomechanical auditory-periphery and auditory-nerve models (57–59), replacing the Gaussian vulnerability kernels with mechanistically derived frequency weightings.

### 6.5 Assumptions and limitations

The framework rests on explicit assumptions that bound its interpretation. (i) It is computational and hypothesis-generating: all results derive from a closed-form model and an *n* = 500 synthetic dataset, with no new audiometric measurements. (ii) The vulnerability kernels *g*_*X*_(*f*) are informed *prior* choices anchored to the cited literature, not parameters fitted to data; their robustness is probed by Monte-Carlo randomization (Section 4.3) rather than estimated with confidence intervals. (iii) The hypoxia term is clipped by the 14 CFR 25.841 cabin-altitude cap, making it near-constant across the commercial cruise envelope, and its kernel is transferred from higher-altitude (5,300 m) data (Section 4.5). (iv) The radiation axis extrapolates a frequency signature from Gy-scale radiotherapy to mSv-scale cruise exposure and is treated as exploratory, with a direct low-dose cochlear evidence base presently absent. (v) The machine-learning step is an internal model-recovery / identifiability check on model-generated labels, not a biological validation. (vi) The framework yields a frequency *prior*; it does not establish that cosmic radiation harms the aircrew cochlea, nor that TSC prevents aircrew hearing loss, both require the prospective evaluation outlined above. None of these limitations is concealed: the design goal is a transparent, falsifiable decision-support prior, not a validated clinical instrument.

## 7 Conclusions

We have introduced and operationalized a transparent, physics- and audiology-informed framework that integrates three mechanism-distinct cochlear stressors of the aircrew operational environment (cabin acoustic noise, hypobaric hypoxia under regulatory cabin-pressure caps, and altitude-dependent cosmic ionizing radiation) into a single frequency-resolved score, the Cumulative Cochlear Stress Index (CCSI). Four conclusions follow.

1. Convergence band. Across the noise, hypoxia and radiation literatures, the dominant frequency band of cochlear vulnerability in aircrew is 3–6 kHz, with peak emphasis shifting modestly across the three pathways but with a robust geometric overlap. This convergence band coincides exactly with the empirical TSC region (3, 4, or 6 kHz) defined by the Kwak & Kwak 2020 randomized controlled trial (35).
2. Model transparency. The CCSI score is a closed-form weighted sum of three physics- and physiology-derived dose proxies, each multiplied by a frequency-resolved Gaussian vulnerability kernel in log-octave space. The radiation component is anchored to a screening-level approximation to the CARI-7 dose model (24) and is benchmarked against published in-flight aircrew dosimetry (27, 38); the hypoxia component is derived from the alveolar gas equation under the FAA cabin-altitude regulatory cap; the noise component is an A-weighted occupational exposure term. No black-box approximation is used.
3. Robustness and sensitivity. A structured sensitivity analysis evaluating the global CCSI argmax over the full audiometric grid F={0.5,1,2,3,4,6,8,12}kHz finds the argmax inside $F_{\text{TSC}}=\left\{3,4,6\right\}$kHz in all 24 profile × weight cells, and a Monte-Carlo randomization of the kernel parameters within literature bounds reproduces the 4 kHz convergence in 99.2% of draws vs. a 23.1% chance baseline, providing a non-tautological robustness check on the convergence claim. A machine-learning identifiability check (Supplementary material) confirms that each input feature activates the band predicted by its underlying physiology; because its regression labels are themselves CCSI values, it is an internal model-recovery check rather than a biological validation; the high *R*^2^ should not be interpreted as evidence of biological validity, or of predictive accuracy for real-world audiometric outcomes.
4. Clinical translation. Under the default literature prior weighting the CCSI argmax within $F_{\text{TSC}}$ falls on 4 kHz for all four representative aircrew profiles, in agreement with the established 4 kHz noise-notch pattern in civilian and military aircrew (1, 2), with 3 kHz emerging as the alternative under a hypoxia-dominant weighting. The framework thereby identifies a candidate profile-specific TSC target that extends the current audiogram-triggered curative use of TSC toward a hypothesis-driven *prospective evaluation* indexed by the operational flight profile.

The framework is designed to support, rather than replace, the prospective audiometric cohort that will ultimately be required to test the convergence hypothesis directly; the explicit cohort design implications are reported in Section 6.

## Data Availability

The original contributions presented in the study are included in the article/Supplementary material, further inquiries can be directed to the corresponding author.

## References

[1] FalcãoTP SchützGE LuizRR. Audiometric profile of civilian pilots according to noise exposure. Rev Saúde P'*ublica*. (2014) 48:790–6. doi: 10.1590/S0034-8910.201404800525625372170 PMC4211569

[2] KuoCY HungCL ChenHC ShihCP LuRH ChenCW . The immediate and long-term impact of military aircraft noise on hearing: a cross-sectional comparison of fighter pilots and ground staff. Int J Environ Res Public Health. (2021) 18:2982. doi: 10.3390/ijerph1806298233799421 PMC7999744

[3] Lindgren T Wieslander G Dammström BG Norbäck D. Hearing status among commercial pilots in a Swedish airline company. Int J Audiol. (2008) 47:515–19. doi: 10.1080/14992020802064633 18608533

[4] Lindgren T Wieslander G Dammström BG Norbäck D. Hearing status among cabin crew in a Swedish commercial airline company. Int Arch Occup Environ Health. (2009) 82:887–92. doi: 10.1007/s00420-008-0372-7 18972126

[5] Wagstaff AS Arva P. Hearing loss in civilian airline and helicopter pilots compared to air traffic control personnel. Aviat Space Environ Med. (2009) 80:857–61. doi: 10.3357/ASEM.1991.2009 19817237

[6] AljaberM AlharbiH. Association of flying time with hearing loss in military pilots. Saudi J Med Med Sci. (2018) 6:155–9. doi: 10.4103/sjmms.sjmms_10_1830787843 PMC6196690

[7] Kuronen P Sorri MJ Pääkkönen R Muhli A. Temporary threshold shift in military pilots measured using conventional and extended high-frequency audiometry after one flight. Int J Audiol. (2003) 42:29–33. doi: 10.3109/14992020309056082 12564513

[8] Mehrparvar AH Mirmohammadi SJ Ghoreyshi A Mollasadeghi A Loukzadeh Z. High-frequency audiometry: a means for early diagnosis of noise-induced hearing loss. Noise Health. (2011) 13:402–6. doi: 10.4103/1463-1741.90295 22122956

[9] U S Government Accountability Office. Commercial Aviation: Pilots and Flight Attendants' Exposure to Noise aboard Aircraft. U.S. GAO (2018). GAO-18-109R. Available online at: https://www.gao.gov/products/gao-18-109r (Accessed July 19, 2026).

[10] FehrenbacherK ApelC BartzN BertschD van der GietM van der GietS . Temporary threshold shift after noise exposure in hypobaric hypoxia at high altitude: results of the ADEMED expedition (2011). Int Arch Occup Environ Health. (2021) 94:1191–9. doi: 10.1007/s00420-021-01715-w34023963 PMC8292300

[11] SainiS SoodA KotwalN KotwalA GuptaTK. A pilot study comparing hearing thresholds of soldiers at induction and after completion of one year in high altitude area. Med J Armed Forces India. (2021) 77:408–12. doi: 10.1016/j.mjafi.2021.04.01034594068 PMC8459120

[12] Attias J Sohmer H Gold S Haran I Shahar A. Noise and hypoxia induced temporary threshold shifts in rats studied by ABR. Hear Res. (1990) 45:247–52. doi: 10.1016/0378-5955(90)90124-8 2162814

[13] Mirza S Richardson H. Otic barotrauma from air travel. J Laryngol Otol. (2005) 119:366–70. doi: 10.1258/0022215053945723 15949100

[14] Stangerup SE Tjernström Ö Klokker M Harcourt J Stokholm J. Point prevalence of barotitis in children and adults after flight, and effect of autoinflation. Aviation, Space, Environ Med. (1998) 69:45–9. 9451533

[15] Miyazawa T Ueda H Yanagita N. Eustachian tube function and middle ear barotrauma associated with extremes in atmospheric pressure. Ann Otol Rhinol Laryngol. (1996) 105:887–92. doi: 10.1177/000348949610501109 8916865

[16] BhattacharyaS SinghA MarzoRR. “Airplane ear”–a neglected yet preventable problem. AIMS Public Health. (2019) 6:320–5. doi: 10.3934/publichealth.2019.3.32031637280 PMC6779601

[17] O'Neill OJ Brett K Frank AJ. Ear Barotrauma. In: StatPearls [Internet]. Treasure Island, FL: StatPearls Publishing (2026). Available online at: https://www.ncbi.nlm.nih.gov/books/NBK499851/ (Accessed July 19, 2026).

[18] International Commission on Radiological Protection. The 2007 recommendations of the international commission on radiological protection. ICRP publication 103. Ann ICRP (2007) 37:1–332. doi: 10.1016/j.icrp.2007.10.003 18082557

[19] PukkalaE AspholmR AuvinenA EliaschH GundestrupM HaldorsenT . Incidence of cancer among Nordic airline pilots over five decades: occupational cohort study. BMJ. (2002) 325:567. doi: 10.1136/bmj.325.7364.56712228131 PMC124549

[20] Pukkala E Helminen M Haldorsen T Hammar N Kojo K Linnersjö A . Cancer incidence among Nordic airline cabin crew. Int J Cancer. (2012) 131:2886–97. doi: 10.1002/ijc.27551 22447246

[21] Langner I Blettner M Gundestrup M Storm H Aspholm R Auvinen A . Cosmic radiation and cancer mortality among airline pilots: results from a European cohort study (ESCAPE). Radiat Environ Biophys. (2004) 42:247–56. doi: 10.1007/s00411-003-0214-7 14648170

[22] Hammer GP Auvinen A De Stavola BL Grajewski B Gundestrup M Haldorsen T . Mortality from cancer and other causes in commercial airline crews: a joint analysis of cohorts from 10 countries. Occup Environ Med. (2014) 71:313–22. doi: 10.1136/oemed-2013-101395 24389960

[23] TopraniSM ScheiblerC MordukhovichI McNeelyE NagelZD. Cosmic ionizing radiation: a DNA-damaging agent that may underly excess cancer in flight crews. Int J Mol Sci. (2024) 25:7670. doi: 10.3390/ijms2514767039062911 PMC11277465

[24] Copeland K. CARI-7A: development and validation. Radiat Prot Dosimetry. (2017) 175:419–31. doi: 10.1093/rpd/ncw369 28074016

[25] MertensCJ MeierMM BrownS NormanRB XuX. NAIRAS aircraft radiation model development, dose climatology, and initial validation. Space Weather. (2013) 11:603–35. doi: 10.1002/swe.2010026213513 PMC4508919

[26] Bottollier-Depois JF Chau Q Bouisset P Kerlau G Plawinski L Lebaron-Jacobs L. Assessing exposure to cosmic radiation during long-haul flights. Radiat Res. (2000) 153:526–32. doi: 10.1667/0033-7587(2000)153[0526:AETCRD]2.0.CO;2 10790273

[27] Lee J Nam UW Pyo J Kim S Kwon YJ Lee J . Short-term variation of cosmic radiation measured by aircraft under constant flight conditions. Space Weather. (2015) 13:797–806. doi: 10.1002/2015SW001288

[28] BhandareN JacksonA EisbruchA PanCC FlickingerJC AntonelliP . Radiation therapy and hearing loss. Int J Radiat Oncol Biol Phys. (2010) 76:S50–7. doi: 10.1016/j.ijrobp.2009.04.09620171518 PMC3319461

[29] Theunissen EAR Bosma SCJ Zuur CL Spijker R van der Baan S Dreschler WA . Sensorineural hearing loss in patients with head and neck cancer after chemoradiotherapy and radiotherapy: a systematic review of the literature. Head Neck. (2015) 37:281–92. doi: 10.1002/hed.23551 24478269

[30] Liu Z Luo Y Guo R Yang B Shi L Sun J . Head and neck radiotherapy causes significant disruptions of cochlear ribbon synapses and consequent sensorineural hearing loss. Radiother Oncol. (2022) 173:207–14. doi: 10.1016/j.radonc.2022.05.023 35640772

[31] NoreñaAJ EggermontJJ. Enriched acoustic environment after noise trauma reduces hearing loss and prevents cortical map reorganization. J Neurosci. (2005) 25:699–705. doi: 10.1523/JNEUROSCI.2226-04.200515659607 PMC6725313

[32] Noreña AJ Eggermont JJ. Enriched acoustic environment after noise trauma abolishes neural signs of tinnitus. Neuroreport. (2006) 17:559–63. doi: 10.1097/00001756-200604240-00001 16603911

[33] RecanzoneGH SchreinerCE MerzenichMM. Plasticity in the frequency representation of primary auditory cortex following discrimination training in adult owl monkeys. J Neurosci. (1993) 13:87–103. doi: 10.1523/JNEUROSCI.13-01-00087.19938423485 PMC6576321

[34] Eggermont JJ. Acquired hearing loss and brain plasticity. Hear Res. (2017) 343:176–90. doi: 10.1016/j.heares.2016.05.008 27233916

[35] Kwak E Kwak S. Threshold sound conditioning in the treatment of sensorineural hearing loss. Laryngosc Investig Otolaryngol. (2020) 5:438–44. doi: 10.1002/lio2.399 PMC7314479 32596485

[36] Cho S Kwak E Kwak S Lopez J. Randomized controlled trial evaluating threshold sound conditioning in the treatment of sensorineural hearing loss (S26.006). Neurology. (2015) 84:S26.006. doi: 10.1212/WNL.84.14_supplement.S26.006

[37] Kwak E Kwak S. Erratum: threshold sound conditioning in the treatment of sensorineural hearing loss (vol 5, pg 438, 2020). Laryngosc Investig Otolaryngol. (2021) 6:1489. doi: 10.1002/lio2.680 PMC7314479 32596485

[38] Lindborg L Bartlett D Beck P McAulay I Schnuer K Schraube H . Cosmic radiation exposure of aircraft crew: compilation of measured and calculated data. Radiat Prot Dosimetry. (2004) 110:417–22. doi: 10.1093/rpd/nch232 15353684

[39] Meier MM Trompier F Ambrozova I Kubancak J Matthiä D Ploc O . CONCORD: comparison of cosmic radiation detectors in the radiation field at aviation altitudes. J Space Weather Space Clim. (2016) 6:A24. doi: 10.1051/swsc/2016017

[40] Breiman L. Random forests. Mach Learn. (2001) 45:5–32. doi: 10.1023/A:1010933404324

[41] Friedman JH. Greedy function approximation: a gradient boosting machine. Ann Stat. (2001) 29:1189–232. doi: 10.1214/aos/1013203451

[42] Pedregosa F Varoquaux G Gramfort A Michel V Thirion B Grisel O . Scikit-learn: machine learning in Python. J Mach Learn Res. (2011) 12:2825–30.

[43] Matthiä D Meier MM Reitz G. Numerical calculation of the radiation exposure from galactic cosmic rays at aviation altitudes with the PANDOCA core model. Space Weather. (2014) 12:161–71. doi: 10.1002/2013SW001022

[44] Bartlett DT. Radiation protection aspects of the cosmic radiation exposure of aircraft crew. Radiat Prot Dosimetry. (2004) 109:349–55. doi: 10.1093/rpd/nch311 15273353

[45] SimonsenLC SlabaTC GuidaP RusekA. NASA's first ground-based galactic cosmic ray simulator: enabling a new era in space radiobiology research. PLoS Biol. (2020) 18:e3000669. doi: 10.1371/journal.pbio.300066932428004 PMC7236977

[46] Cucinotta FA Alp M Sulzman FM Wang M. Space radiation risks to the central nervous system. Life Sci Space Res. (2014) 2:54–69. doi: 10.1016/j.lssr.2014.06.003

[47] Bhandare N Antonelli PJ Morris CG Malayapa RS Mendenhall WM. Ototoxicity after radiotherapy for head and neck tumors. Int J Radiat Oncol Biol Phys. (2007) 67:469–79. doi: 10.1016/j.ijrobp.2006.09.017 17236969

[48] Mujica-Mota MA Lehnert S Devic S Gasbarrino K Daniel SJ. Mechanisms of radiation-induced sensorineural hearing loss and radioprotection. Hear Res. (2014) 312:60–68. doi: 10.1016/j.heares.2014.03.003 24650954

[49] ShiW HouX BaoX HouW JiangX MaL . Mechanism and protection of radiotherapy-induced sensorineural hearing loss for head and neck cancer. Biomed Res Int. (2021) 2021:3548706. doi: 10.1155/2021/354870634970625 PMC8714384

[50] KujawaSG LibermanMC. Adding insult to injury: cochlear nerve degeneration after “temporary” noise-induced hearing loss. J Neurosci. (2009) 29:14077–85. doi: 10.1523/JNEUROSCI.2845-09.200919906956 PMC2812055

[51] LibermanMC EpsteinMJ ClevelandSS WangH MaisonSF. Toward a differential diagnosis of hidden hearing loss in humans. PLoS ONE. (2016) 11:e0162726. doi: 10.1371/journal.pone.016272627618300 PMC5019483

[52] BrozoskiTJ SpiresTJD BauerCA. Vigabatrin, a GABA transaminase inhibitor, reversibly eliminates tinnitus in an animal model. J Assoc Res Otolaryngol. (2007) 8:105–18. doi: 10.1007/s10162-006-0067-217221143 PMC2538419

[53] TylerRS CoelhoC TaoP JiH NobleW GehringerA . Identifying tinnitus subgroups with cluster analysis. Am J Audiol. (2008) 17:S176–84. doi: 10.1044/1059-0889(2008/07-0044)19056922 PMC2668860

[54] Kwak S. Method and Device for Objective Automated Audiometry. US Patent No 8,177,727 (Earlogic Korea, Inc. and Sangyeop Kwak), granted 15 May 2012 (2012).

[55] Lee D Kwak S Jang S. Method and Apparatus for Hearing Improvement Based on Cochlear Model. US Patent No 11,070,924 (GoldenEar Company, Inc., Valencia, CA), granted 20 July 2021 (2021).

[56] Kwak S Lee D Jang S Kim S Kim S Doo W . Stochastic modeling of tinnitus loudness. bioRxiv. (2023). doi: 10.1101/2023.02.09.527783

[57] Verhulst S Altoe A Vasilkov V. Computational modeling of the human auditory periphery: auditory-nerve responses, evoked potentials and hearing loss. Hear Res. (2018) 360:55–75. doi: 10.1016/j.heares.2017.12.018 29472062

[58] ZilanyMSA BruceIC CarneyLH. Updated parameters and expanded simulation options for a model of the auditory periphery. J Acoust Soc Am. (2014) 135:283–6. doi: 10.1121/1.483781524437768 PMC3985897

[59] Carney LH. A model for the responses of low-frequency auditory-nerve fibers in cat. J Acoust Soc Am. (1993) 93:401–17. doi: 10.1121/1.405620 8423257

