# Supplementary material for: Convergent 3–6 kHz cochlear vulnerability in aircrew: matching threshold sound conditioning targets to operational flight profiles
Source: Front Neurol. 2026 Jul 28;17:1903374. doi: 10.3389/fneur.2026.1903374 (PMC13494270; doi:10.3389/fneur.2026.1903374)
Supplement: Supplementary file 1 [file Supplementary_file_1.pdf]

Supplementary Material

This document supports the main manuscript. It provides the computational framework details (S1), the machine-learning identifiability check and Supplementary Figure S1 (S2), the complete released Python source code (S3), and the released data-file manifest (S4). All materials are also deposited on Zenodo (DOI assigned upon acceptance). Labels prefixed with "S" refer to this supplement; all other references are to the main text.

S1 COMPUTATIONAL FRAMEWORK DETAILS

S1.1 Frequency-resolved vulnerability kernels

Each mechanism-distinct kernel is a Gaussian in log-octave frequency space,

$$g_X(f) = \exp\Big[-\tfrac{1}{2}\big(\log_2(f/\mu_X)/\sigma_X\big)^2\Big], \qquad X \in \{N, H, R\}, \tag{1}$$

with the literature-anchored parameters of Table S1. These are informed prior choices, not fitted parameters; their robustness is quantified by the Monte-Carlo test of Section S1.3.

Table S1: Vulnerability-kernel parameters.

Kernel	Pathway	μ_X (kHz)	σ_X (oct)
g_N	Cabin acoustic noise	4	0.8
g_H	Hypobaric hypoxia	3	1.0
g_R	Ionizing radiation (explor.)	6	0.9

S1.2 Physics dose proxies and normalizations

The per-segment hazard doses are the radiation dose $D_R(h, R_c, \mathrm{HCP}; t)$ (screening-level closed-form approximation to FAA CARI-7, benchmarked against the in-flight Liulin measurement of Lee et al. 2015), the hypoxia dose $D_H(h; t)$ (simplified West alveolar gas equation under the 14 CFR 25.841 cabin-altitude cap, normalized so one unit = an 8 h flight at the cap-equivalent hypoxia index $H_{\mathrm{cap}} \approx 0.45$), and the noise dose $D_N(L_{Aeq}; t)$ (A-weighted occupational term above a 65 dBA floor, one unit = an 8 h segment at 95 dBA). Full definitions are in the main text; the reference implementation is `analysis_main.py` (Section S3).

S1.3 Anti-tautology Monte-Carlo test

We randomized the kernel parameters ($N = 2 \times 10^5$ draws) and recorded how often the discrete-grid argmax of the geometric-mean convergence index falls inside $\mathcal{F}_{\mathrm{TSC}} = \{3, 4, 6\}$ kHz (Table S2). Under mechanistic uncertainty the 4 kHz convergence is reproduced in 99.2% of draws; under an unconstrained null it occurs in only 23.1%, a 4.3× enrichment. The routine is `null_test.py` (Section S3).

Table S2: Monte-Carlo robustness of the convergence argmax ($N = 2 \times 10^5$, seed 42). Mechanistic ranges: $\mu_N \in [3.5, 4.5]$, $\mu_H \in [2.5, 3.5]$, $\mu_R \in [5, 7]$ kHz, $\sigma_X \in [0.7, 1.1]$ oct. Unconstrained null: all peaks log-uniform over 1–8 kHz.

Scenario	$P(\arg\max \in \mathcal{F}_{\text{TSC}})$	$P(\arg\max = 4\,\text{kHz})$
Mechanistic uncertainty	100.0%	99.2%
Unconstrained null	64.6%	23.1%

S2 MACHINE-LEARNING IDENTIFIABILITY CHECK

The machine-learning analysis reported here is an *internal model-recovery and identifiability check*, not a biological validation, and is therefore presented as supplementary material. Its sole purpose is to confirm that each operational input activates the frequency bands predicted by its underlying physiology, given that the regression targets are themselves the closed-form CCSI values defined in the main text.

S2.1 Pipeline

For each frequency band f in the audiometric grid $\{0.5, 1, 2, 3, 4, 6, 8, 12\}$ kHz we trained three regression models on the seven engineered features $(h, t, R_c, \text{HCP}, L_{Aeq}, H, D_R)$ standardized to zero mean and unit variance: Random Forest (n_{est}=100, default tree depth, random state 42), Gradient Boosting (n_{est}=100, learning rate 0.1), and Ridge regression (α=1.0). Performance was evaluated by 5-fold cross-validation on the R^2 and mean-absolute-error metrics. Feature importance for the tree-based models was extracted as impurity-based importance (mean decrease in the squared-error splitting criterion), and for Ridge from the absolute standardized coefficients. Python 3.10 with scikit-learn 1.3.

S2.2 Results

Random Forest, Gradient Boosting and Ridge converge to a band-averaged 5-fold cross-validated R^2 of 0.981, 0.982 and 0.978, respectively, with mean absolute errors near 0.009–0.011 in CCSI units (Supplementary Figure S1A). These are *model-recovery* metrics: because the labels are themselves CCSI values, the ensembles recover the closed-form mapping rather than predicting an independently measured biological outcome; the high R^2 reflects internal model consistency and feature-band identifiability, not biological validation. For the 4 kHz label, flight-hours t accounts for $\approx 94.0\%$ of the Random-Forest importance, noise L_{Aeq} for $\approx 4.8\%$, and the remaining five features $(h, R_c, \text{HCP}, H, D_R)$ together for $\approx 1.2\%$ (Supplementary Figure S1B); the flight-hours dominance is a direct consequence of the linear time-scaling of the dose proxies. The row-normalized feature-importance heatmap (Supplementary Figure S1C) shows that each feature activates the bands predicted by its physiology: L_{Aeq} peaks at 3–6 kHz, D_R peaks at 4–8 kHz, R_c and HCP co-localize with the radiation pattern, and altitude h shows residual importance at the low-frequency end. This feature-band signature is consistent with the CCSI construction by design and should not be read as independent biological evidence.

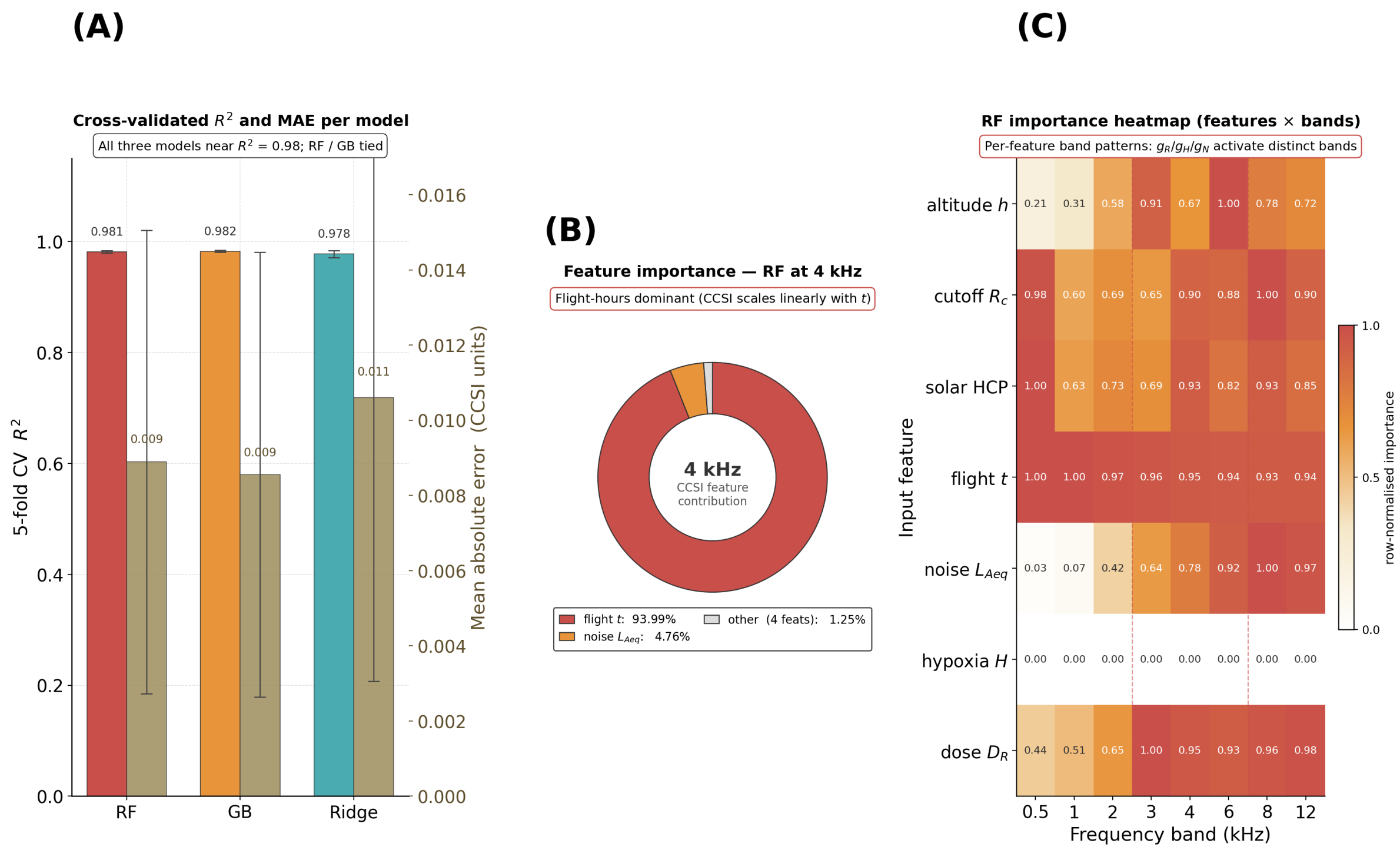

Figure S1: **ML ensemble performance and feature interpretation (identifiability check).** (A) Band-averaged 5-fold cross-validated R^2 and mean absolute error for Random Forest, Gradient Boosting and Ridge. (B) Random-Forest impurity-based feature importance for the 4 kHz CCSI prediction. (C) Row-normalized feature-importance heatmap across all eight bands; dashed verticals delimit the 3–6 kHz convergence band.

S3 PYTHON SOURCE CODE

The complete analysis is released as two self-contained scripts that reproduce every figure, table and reported statistic. Python 3.10 with `numpy`, `pandas`, `scikit-learn 1.3` and `matplotlib`.

S3.1 `analysis_main.py` — CCSI, figures, ML, sensitivity

```
"""
analysis_main.py
=================
CCSI (Cumulative Cochlear Stress Index) -- computational framework for
frequency-resolved auditory vulnerability prediction in aircrew.

Paper: "Convergent 3-6 kHz cochlear vulnerability in aircrew: matching
threshold sound conditioning targets to operational flight profiles"
Target journal: Frontiers in Neurology (Neuro-Otology), Hypothesis & Theory
Authors: D. Lee, S. Jang, K. Kang, E. Choi, C.-M. Yang, J. Yoo, S. Kim,
         S. Kwak, D.-H. Lee (corresponding)

Pipeline:
  1) Synthetic dataset generator (n=500) parametrised over altitude, latitude
     (Rc), heliocentric potential (HCP), flight-hours, cabin noise.
  2) Physics-based hazard dose proxies:
       - D_radiation : screening-level exponential approximation to the
                       FAA CARI-7 model, extended with latitude correction (Tier 1.5).
       - D_hypoxia   : Alveolar gas equation-based PaO2 deficit relative to
                       sea level.
       - D_noise     : A-weighted equivalent dose with octave-band weighting.
  3) Frequency-dependent vulnerability kernels g_R(f), g_H(f), g_N(f).
  4) CCSI(f) = w_R * D_R * g_R(f) + w_N * D_N * g_N(f) + w_H * D_H * g_H(f)
  5) ML ensemble (Random Forest, Gradient Boosting, Ridge) trained on
     CCSI labels with Gaussian noise; 5-fold CV + bootstrap (B=1000).
  6) Output: synthetic_dataset.csv, experiment_summary.json, 5 figures.

Figure style: per project_instructions GitHub spec
  - figsize=(20,10), dpi=200, hspace=0.55, wspace=0.38
  - Panel labels: 28pt bold at ax.text(0.0, 1.18, '(A)', ...) clip_on=False
  - Warming muted earthtone palette only; cold-blue (#1f77b4, #2C7BB6) banned.
  - facecolor='white', grid #DDDDDD dashed; no top/right spine.

Build date: 2026-05-21
"""

from __future__ import annotations

import json
import os
from dataclasses import dataclass
from pathlib import Path

import matplotlib
import matplotlib.pyplot as plt
import numpy as np
import pandas as pd
from sklearn.ensemble import GradientBoostingRegressor, RandomForestRegressor
from sklearn.linear_model import Ridge
from sklearn.metrics import mean_absolute_error, r2_score
from sklearn.model_selection import KFold, cross_val_score
from sklearn.preprocessing import StandardScaler

# ---------------------------------------------------------------------------
# Global figure style (project_instructions GitHub spec)
# ---------------------------------------------------------------------------
PALETTE = {
    "red":    "#C94F4A",   # Warming muted earth -- primary
    "orange": "#E8943A",   # Warming amber
    "teal":   "#4AACB0",   # Muted teal (allowed; not cold blue)
    "blue":   "#5B8DB8",   # Muted slate-blue (allowed; not cold blue)
    "olive":  "#9C8C5C",   # Earth olive -- accent
```

```
    "rust":    "#A05A2C",    # Rust accent
    "grey":    "#666666",
    "lightgrey": "#DDDDDD",
}

matplotlib.rcParams.update({
    "font.family": "DejaVu Sans",
    "font.size": 13,
    "axes.titlesize": 16,
    "axes.labelsize": 16,
    "xtick.labelsize": 15,
    "ytick.labelsize": 15,
    "legend.fontsize": 13,
    "axes.spines.top": False,
    "axes.spines.right": False,
    "axes.linewidth": 0.9,
    "axes.facecolor": "white",
    "figure.facecolor": "white",
    "grid.color": PALETTE["lightgrey"],
    "grid.linestyle": "--",
    "grid.linewidth": 0.6,
    "savefig.dpi": 200,
    "savefig.bbox": "tight",
    "savefig.pad_inches": 0.2,
})


def add_panel_label(ax, label: str) -> None:
    """Panel label per project_instructions: ax.text(0.0, 1.18, ...) 28pt bold."""
    ax.text(
        0.0, 1.18, label,
        transform=ax.transAxes,
        ha="left", va="bottom",
        fontsize=28, fontweight="bold",
        clip_on=False,
    )


def style_axis(ax, title: str | None = None) -> None:
    ax.set_facecolor("white")
    ax.grid(True, color=PALETTE["lightgrey"], ls="--", lw=0.6, alpha=0.9)
    ax.set_axisbelow(True)
    if title:
        ax.set_title(title, pad=6, fontsize=16)


# ============================================================================
# 1) Physics-based dose proxies
# ============================================================================

def dose_radiation_per_hour(altitude_km: np.ndarray,
                            Rc_GV: np.ndarray,
                            HCP_MV: np.ndarray) -> np.ndarray:
    """
    Effective dose rate (uSv/h) -- screening-level exponential approximation
    to the FAA CARI-7 model, extended with latitude correction (Tier 1.5):

       D(h, Rc, HCP) = 0.0424 * exp(0.335*h) * f_lat(Rc) * f_sol(HCP)

    where the Rc-saturation latitude factor is anchored to Korean Peninsula
    Rc_ref = 9.96 GV (a = 0.0994, n_hat = 1.289), and solar modulation
    f_sol scales linearly to give +13% at HCP_min.
    """
    Rc_ref = 9.96
    a = 0.0994
    n = 1.289
    HCP_max = 1141.0
    HCP_min = 270.0

    f_lat = (1 + a * Rc_ref ** n) / (1 + a * Rc_GV ** n)
```

```
    f_sol = 1 + 0.13 * (HCP_max - HCP_MV) / (HCP_max - HCP_min)

    D = 0.0424 * np.exp(0.335 * altitude_km) * f_lat * f_sol
    return D  # uSv/h


def cabin_altitude_from_cruise(altitude_km: np.ndarray) -> np.ndarray:
    """
    Approximate cabin-altitude pressurisation schedule for commercial aviation.
    Below 6 km: ambient. Above 6 km: limit to FAA 14 CFR 25.841 (~2.44 km, 8000 ft).
    Linear blend below cruise cap.
    """
    cabin_alt_max = 2.44  # km (~8000 ft regulatory cap)
    return np.minimum(altitude_km, cabin_alt_max)


def PaO2_at_altitude(cabin_altitude_km: np.ndarray) -> np.ndarray:
    """
    Alveolar gas equation (simplified West).
       PaO2 ~ FiO2 * (P_atm - 47) - PaCO2/RQ
    with P_atm(h) = 760 * exp(-h/7.4)  mmHg (h in km),
    FiO2 = 0.2095, PaCO2 = 40 mmHg, RQ = 0.8 (resting healthy adult).
    """
    P_atm = 760.0 * np.exp(-cabin_altitude_km / 7.4)
    FiO2 = 0.2095
    PaCO2 = 40.0
    RQ = 0.8
    return FiO2 * (P_atm - 47.0) - PaCO2 / RQ


H_CAP = 0.45  # cap-altitude hypoxia index ~ 1 - 55/99 under FAA 25.841 (2.44 km)


def hypoxia_index(altitude_km: np.ndarray) -> np.ndarray:
    """
    Hypoxia index = 1 - PAO2(h)/PAO2(0). Range ~0..0.45 at cabin alt 2.44 km
    (alveolar gas equation; arterial PaO2 differs by A-a gradient).
    """
    cabin = cabin_altitude_from_cruise(altitude_km)
    PaO2 = PaO2_at_altitude(cabin)
    PaO2_0 = PaO2_at_altitude(np.array([0.0]))[0]
    return np.clip(1 - PaO2 / PaO2_0, 0, 1)


def cabin_noise_LAeq_dB(altitude_km: np.ndarray,
                        rng: np.random.Generator) -> np.ndarray:
    """
    Empirical cabin noise (LAeq, dBA) as function of cruise altitude.
    Anchored to GAO 2018: 74-84 dBA in cabins; cockpit 75-81 dBA, fighter 85-95.
    Slight positive trend with altitude (engine throttle + thinner air noise).
    """
    base = 74.0 + 0.9 * altitude_km  # mild positive trend
    noise = rng.normal(0, 2.0, size=altitude_km.shape)  # population variance
    return np.clip(base + noise, 60, 100)


# ============================================================================
# 2) Frequency-dependent vulnerability kernels
# ============================================================================

FREQ_BANDS_HZ = np.array([500, 1000, 2000, 3000, 4000, 6000, 8000, 12000])
FREQ_BANDS_KHZ = FREQ_BANDS_HZ / 1000.0


def gaussian_kernel(freq_khz: np.ndarray, peak_khz: float, sigma_oct: float) -> np.ndarray:
    """Gaussian in log2 (octave) space, peak at peak_khz, width sigma_oct."""
    log_diff = np.log2(freq_khz / peak_khz)
    return np.exp(-0.5 * (log_diff / sigma_oct) ** 2)
```

```
def vulnerability_noise(freq_khz: np.ndarray) -> np.ndarray:
    """
    Noise-induced vulnerability -- classical 4 kHz notch (Falcao 2014: peak 4 kHz
    20.6%, 6 kHz 16.9%, 3 kHz 10.5%). Gaussian peak at 4 kHz, sigma 0.8 octave.
    """
    return gaussian_kernel(freq_khz, peak_khz=4.0, sigma_oct=0.8)


def vulnerability_radiation(freq_khz: np.ndarray) -> np.ndarray:
    """
    Radiation-induced cochlear vulnerability -- high-frequency bias (Liu 2022:
    ribbon-synapse damage selective for basal cochlea / high-freq region).
    Gaussian peak at 6 kHz, sigma 0.9 octave.
    """
    return gaussian_kernel(freq_khz, peak_khz=6.0, sigma_oct=0.9)


def vulnerability_hypoxia(freq_khz: np.ndarray) -> np.ndarray:
    """
    Hypobaric hypoxia vulnerability -- Pollok/Fehrenbacher ADEMED + Singh:
    threshold shift maxima at 2-4 kHz with broader plateau than NIHL.
    Gaussian peak at 3 kHz, sigma 1.0 octave.
    """
    return gaussian_kernel(freq_khz, peak_khz=3.0, sigma_oct=1.0)


# ============================================================================
# 3) CCSI computation
# ============================================================================

@dataclass
class CCSIWeights:
    w_R: float = 0.35   # radiation contribution weight (per uSv/h-hour-equiv unit)
    w_N: float = 0.45   # noise contribution weight
    w_H: float = 0.20   # hypoxia contribution weight

    def normalised(self) -> "CCSIWeights":
        s = self.w_R + self.w_N + self.w_H
        return CCSIWeights(self.w_R / s, self.w_N / s, self.w_H / s)


def compute_CCSI(altitude_km: np.ndarray,
                 flight_hours: np.ndarray,
                 Rc_GV: np.ndarray,
                 HCP_MV: np.ndarray,
                 noise_dBA: np.ndarray,
                 weights: CCSIWeights | None = None,
                 freq_khz: np.ndarray = FREQ_BANDS_KHZ) -> np.ndarray:
    """
    Cumulative Cochlear Stress Index (frequency-resolved).
    Returns array shape (n_samples, n_freq).
    Units: dimensionless (normalised stress score).
    """
    w = (weights or CCSIWeights()).normalised()

    # Per-flight hazard "doses" (accumulated over flight_hours)
    D_R = dose_radiation_per_hour(altitude_km, Rc_GV, HCP_MV) * flight_hours
    # Normalise D_R: typical aircrew 600 h/yr x 1.4 uSv/h = 840 uSv/yr ~ 1 unit
    D_R_norm = D_R / 1000.0

    # Noise dose: A-weighted equivalent -- log-linear from 65 dBA baseline
    D_N = np.maximum(0, noise_dBA - 65.0) / 30.0 * flight_hours / 8.0
    # Normalise: 8h/day at 95 dBA -> 1 unit

    # Hypoxia: integrated hypoxia_index over flight_hours
    H_idx = hypoxia_index(altitude_km)
    # Normalisation: 1 unit corresponds to 8 h at the cap-altitude hypoxia
    # index H_CAP ~ 0.45 (alveolar gas equation under FAA 25.841 cap).
    D_H_norm = (H_idx * flight_hours / 8.0) / H_CAP
```

```python
    # Frequency-dependent vulnerability kernels
    g_R = vulnerability_radiation(freq_khz)   # shape (n_freq,)
    g_N = vulnerability_noise(freq_khz)
    g_H = vulnerability_hypoxia(freq_khz)

    # Broadcast: CCSI shape (n_samples, n_freq)
    CCSI = (
        w.w_R * D_R_norm[:, None] * g_R[None, :]
        + w.w_N * D_N[:, None] * g_N[None, :]
        + w.w_H * D_H_norm[:, None] * g_H[None, :]
    )
    return CCSI


# ==============================================================================
# 4) Synthetic dataset generator
# ==============================================================================

def generate_synthetic_dataset(n: int = 500, seed: int = 42) -> pd.DataFrame:
    """
    Generate n synthetic aircrew flight profiles.
    Sampled uniformly over plausible operational ranges.
    """
    rng = np.random.default_rng(seed)

    altitude = rng.uniform(3.0, 12.2, size=n)            # km (3-12.2 = ~10k-40k ft)
    flight_hours = rng.uniform(1.0, 12.0, size=n)         # per flight
    Rc = rng.uniform(0.5, 17.0, size=n)                   # geomagnetic cutoff GV
    HCP = rng.uniform(270.0, 1141.0, size=n)              # solar modulation MV
    noise = cabin_noise_LAeq_dB(altitude, rng)            # dBA, depends on alt

    df = pd.DataFrame({
        "altitude_km": altitude,
        "flight_hours": flight_hours,
        "Rc_GV": Rc,
        "HCP_MV": HCP,
        "noise_dBA": noise,
        "cabin_altitude_km": cabin_altitude_from_cruise(altitude),
        "hypoxia_index": hypoxia_index(altitude),
        "dose_radiation_uSv_per_h": dose_radiation_per_hour(altitude, Rc, HCP),
    })

    # Add CCSI labels per frequency band
    CCSI = compute_CCSI(altitude, flight_hours, Rc, HCP, noise)
    for i, f_khz in enumerate(FREQ_BANDS_KHZ):
        # Add 5% Gaussian noise (sklearn benchmark precedent)
        label = CCSI[:, i] * (1 + rng.normal(0, 0.05, size=n))
        df[f"CCSI_{int(FREQ_BANDS_HZ[i])}Hz"] = label

    return df


# ==============================================================================
# 5) Figure 1 -- Multi-hazard overview
# ==============================================================================

def fig01_multihazard(df: pd.DataFrame, out_path: Path) -> None:
    """
    Fig 1.  Multi-hazard aviation auditory environment.
    (A) Altitude vs cosmic radiation dose rate (Tier 1.5 formula curve).
    (B) Altitude vs cabin LAeq noise (scatter + LOWESS trend band).
    (C) Altitude vs hypoxia index (FAA cabin-altitude cap visible).
    """
    fig, axes = plt.subplots(1, 3, figsize=(20, 10),
                             gridspec_kw=dict(wspace=0.38, hspace=0.55))

    # ----- Panel A: Cosmic radiation -----
    ax = axes[0]
    style_axis(ax, title="Cosmic radiation dose rate vs cruise altitude")
    add_panel_label(ax, "(A)")
```

```
h_grid = np.linspace(0, 15, 200)
Rc_polar = np.full_like(h_grid, 1.0)    # polar route
Rc_mid = np.full_like(h_grid, 9.96)     # Korea (reference)
Rc_eq = np.full_like(h_grid, 17.0)      # equatorial
HCP_max = np.full_like(h_grid, 1141.0)  # solar max

D_polar = dose_radiation_per_hour(h_grid, Rc_polar, HCP_max)
D_mid = dose_radiation_per_hour(h_grid, Rc_mid, HCP_max)
D_eq = dose_radiation_per_hour(h_grid, Rc_eq, HCP_max)

ax.plot(h_grid, D_polar, color=PALETTE["red"], lw=2.4,
        label=r"Polar (R$_c$=1.0 GV)")
ax.plot(h_grid, D_mid, color=PALETTE["orange"], lw=2.4,
        label=r"Mid-lat. Korea (R$_c$=9.96 GV)")
ax.plot(h_grid, D_eq, color=PALETTE["teal"], lw=2.4,
        label=r"Equatorial (R$_c$=17.0 GV)")

# Anchor points from Lee 2015 (Korea FL300, ~2.3 +/- 0.17 H*(10) uSv/h)
# Convert H*(10) -> E by /1.5
ax.errorbar([9.144], [2.3 / 1.5], yerr=[0.17 / 1.5], fmt='s',
            color=PALETTE["rust"], markersize=10, capsize=4, lw=1.4,
            label="Lee et al. 2015 (FL300, Korea)", zorder=5)

ax.set_xlabel("Cruise altitude (km)")
ax.set_ylabel(r"Effective dose rate ($\mu$Sv h$^{-1}$)")
ax.set_xlim(0, 15)
ax.set_ylim(0, 5.5)
ax.legend(loc="upper left", frameon=True, framealpha=0.95,
          edgecolor="#333333", facecolor="white")

# ----- Panel B: Cabin noise -----
ax = axes[1]
style_axis(ax, title="Cabin acoustic environment (LAeq)")
add_panel_label(ax, "(B)")

ax.scatter(df["altitude_km"], df["noise_dBA"],
           c=PALETTE["orange"], s=22, alpha=0.55,
           edgecolors=PALETTE["rust"], linewidth=0.3, label="Synthetic n=500")

# Trend line
sort_idx = np.argsort(df["altitude_km"].values)
h_sorted = df["altitude_km"].values[sort_idx]
n_sorted = df["noise_dBA"].values[sort_idx]
window = 25
n_smooth = pd.Series(n_sorted).rolling(window, center=True, min_periods=5).mean()
ax.plot(h_sorted, n_smooth, color=PALETTE["red"], lw=2.6,
        label="Moving avg (w=25)")

# OSHA / NIOSH reference bands -- labels in legend instead of inline
ax.axhline(85, color=PALETTE["grey"], lw=1.2, ls=":", alpha=0.8,
           label="NIOSH REL 85 dBA")
ax.axhline(90, color="#A05A2C", lw=1.2, ls=":", alpha=0.8,
           label="OSHA PEL 90 dBA")

ax.set_xlabel("Cruise altitude (km)")
ax.set_ylabel(r"Cabin noise LA$_{eq}$ (dBA)")
ax.set_xlim(0, 13)
ax.set_ylim(60, 100)
ax.legend(loc="lower right", frameon=True, framealpha=0.95,
          edgecolor="#333333", facecolor="white")

# ----- Panel C: Hypoxia (pressurised vs unpressurised) -----
ax = axes[2]
style_axis(ax, title="Hypobaric hypoxia index: pressurised vs unpressurised")
add_panel_label(ax, "(C)")

h_grid2 = np.linspace(0, 15, 300)
HI_press = hypoxia_index(h_grid2)
# Unpressurised: PaO2 follows ambient barometric pressure throughout
```

```
    PaO2_amb = PaO2_at_altitude(h_grid2)
    PaO2_0 = PaO2_at_altitude(np.array([0.0]))[0]
    HI_unp = np.clip(1 - PaO2_amb / PaO2_0, 0, 1)

    ax.plot(h_grid2, HI_unp, color=PALETTE["red"], lw=2.8,
            label="Unpressurised (ambient)")
    ax.plot(h_grid2, HI_press, color=PALETTE["teal"], lw=2.8,
            label="Pressurised (FAA cap)")
    ax.fill_between(h_grid2, HI_press, HI_unp, where=(HI_unp > HI_press),
                    color=PALETTE["orange"], alpha=0.18,
                    label="Pressurisation benefit")

    # Cabin altitude cap line -- no boxed annotation inside axes
    cap_h = 2.44
    ax.axvline(cap_h, color=PALETTE["grey"], lw=1.0, ls="--", alpha=0.6)

    # Mark cruise altitude operational range
    ax.axvspan(9.1, 12.2, color=PALETTE["orange"], alpha=0.10)

    ax.set_xlabel("Cruise altitude (km)")
    ax.set_ylabel(r"Hypoxia index  $1 - P_{a}O_2(h)/P_{a}O_2(0)$")
    ax.set_xlim(0, 15)
    ax.set_ylim(0, 1.05)
    ax.legend(loc="lower right", frameon=True, framealpha=0.95,
              edgecolor="#333333", facecolor="white")

    # Annotation BOXES above the data axes (per project_instructions:
    # box-in-axes annotations are forbidden; place above (top))
    ax.text(0.50, 1.035, "FAA 14 CFR 25.841 cabin-alt cap (8000 ft)",
            transform=ax.transAxes, ha="center", va="bottom",
            fontsize=11,
            bbox=dict(facecolor="white", edgecolor="#333333",
                      boxstyle="round,pad=0.35", linewidth=0.8))

    plt.savefig(out_path)
    plt.close(fig)
    print(f"  saved {out_path.name}")


# ============================================================================
# 6) Figure 2 -- Frequency-resolved 3-6 kHz convergence
# ============================================================================

# Literature-based prevalence/threshold data (synthesized from cited sources)

# Aircrew NIHL prevalence per frequency (%) -- Falcao TP, Schutz GE, Luiz RR
# (Rev Saude Publica 2014;48:790, n=3,130 Brazilian civilian pilots).
# Values averaged across left/right ears from published values.
AIRCREW_NIHL_PREV = {
    500:   2.5,
    1000:  3.4,
    2000:  5.8,
    3000:  9.5,
    4000: 18.9,
    6000: 15.8,
    8000: 11.7,
    12000: 6.2,
}

# RT-SNHL prevalence per frequency (%) -- literature synthesis from Bhandare 2010
# QUANTEC review and Theunissen 2015 systematic review for head/neck patients
# receiving >45 Gy cochlear dose.  High-frequency biased.
RTSNHL_PREV = {
    500:   8.0,
    1000: 14.0,
    2000: 22.0,
    3000: 32.0,
    4000: 45.0,
    6000: 58.0,
    8000: 67.0,
```

```python
    12000: 72.0,
}


def fig02_frequency_convergence(out_path: Path) -> None:
    """
    Fig 2.  Frequency-resolved auditory-vulnerability convergence at 3–6 kHz.
    (A) Aircrew NIHL prevalence vs frequency (Falcao 2014; n=3,130 pilots).
    (B) Radiotherapy-induced SNHL prevalence vs frequency (Bhandare 2010 /
        Theunissen 2015; cochlear dose >45 Gy).
    (C) Normalised vulnerability kernels (noise/radiation/hypoxia) overlaid
        with the threshold-sound-conditioning (TSC) region (Kwak & Kwak 2020).
    """
    fig, axes = plt.subplots(1, 3, figsize=(20, 10),
                             gridspec_kw=dict(wspace=0.38, hspace=0.55))

    # Common frequency axis on log scale
    freqs_hz = np.array(list(AIRCREW_NIHL_PREV.keys()))
    freqs_khz = freqs_hz / 1000.0

    # ---------- Panel A: Aircrew NIHL prevalence ----------
    ax = axes[0]
    style_axis(ax)
    ax.set_title("Aircrew NIHL prevalence (civilian pilots)",
                 fontsize=13.5, fontweight="bold", pad=28)
    add_panel_label(ax, "(A)")

    prev = np.array([AIRCREW_NIHL_PREV[f] for f in freqs_hz])
    bar_colors = [PALETTE["red"] if 3000 <= f <= 6000 else PALETTE["orange"]
                  for f in freqs_hz]
    bars = ax.bar(np.arange(len(freqs_hz)), prev,
                  color=bar_colors, edgecolor=PALETTE["rust"], linewidth=0.6,
                  width=0.72)
    for bar, v in zip(bars, prev):
        ax.text(bar.get_x() + bar.get_width() / 2,
                bar.get_height() + 0.6, f"{v:.1f}",
                ha="center", va="bottom", fontsize=10, color="#333333")
    ax.set_xticks(np.arange(len(freqs_hz)))
    ax.set_xticklabels([f"{int(f / 1000)}" if f >= 1000 else f"{f/1000:.1f}"
                        for f in freqs_hz])
    ax.set_xlabel("Frequency (kHz)")
    ax.set_ylabel("NIHL prevalence (%)")
    ax.set_ylim(0, 25)
    band_left = list(freqs_hz).index(3000) - 0.5
    band_right = list(freqs_hz).index(6000) + 0.5
    ax.axvspan(band_left, band_right, color=PALETTE["red"], alpha=0.08)
    ax.text(0.50, 1.008, "3–6 kHz convergence band",
            transform=ax.transAxes, ha="center", va="bottom",
            fontsize=11,
            bbox=dict(facecolor="white", edgecolor=PALETTE["red"],
                      boxstyle="round,pad=0.35", linewidth=1.0))

    # ---------- Panel B: RT-SNHL prevalence ----------
    ax = axes[1]
    style_axis(ax)
    ax.set_title(r"Radiotherapy-induced SNHL ($>$45 Gy cochlear)",
                 fontsize=13.5, fontweight="bold", pad=28)
    add_panel_label(ax, "(B)")

    prev_rt = np.array([RTSNHL_PREV[f] for f in freqs_hz])
    bar_colors_b = [PALETTE["red"] if 3000 <= f <= 6000 else PALETTE["teal"]
                    for f in freqs_hz]
    bars = ax.bar(np.arange(len(freqs_hz)), prev_rt,
                  color=bar_colors_b, edgecolor="#2A6D70", linewidth=0.6,
                  width=0.72)
    for bar, v in zip(bars, prev_rt):
        ax.text(bar.get_x() + bar.get_width() / 2,
                bar.get_height() + 1.3, f"{v:.0f}",
                ha="center", va="bottom", fontsize=10, color="#333333")
    ax.set_xticks(np.arange(len(freqs_hz)))
```

```
    ax.set_xticklabels([f"{int(f / 1000)}" if f >= 1000 else f"{f/1000:.1f}"
                        for f in freqs_hz])
    ax.set_xlabel("Frequency (kHz)")
    ax.set_ylabel("RT-SNHL prevalence (%)")
    ax.set_ylim(0, 85)
    ax.axvspan(band_left, band_right, color=PALETTE["red"], alpha=0.08)
    ax.text(0.50, 1.008, "High-freq bias  +  3-6 kHz overlap with (A)",
            transform=ax.transAxes, ha="center", va="bottom",
            fontsize=11,
            bbox=dict(facecolor="white", edgecolor=PALETTE["red"],
                      boxstyle="round,pad=0.35", linewidth=1.0))

    # ---------- Panel C: Normalised vulnerability kernels + TSC region ----------
    ax = axes[2]
    style_axis(ax)
    ax.set_title("Normalized vulnerability kernels and TSC region",
                 fontsize=13.5, fontweight="bold", pad=28)
    add_panel_label(ax, "(C)")

    f_fine_khz = np.logspace(np.log10(0.5), np.log10(12), 300)
    g_N = vulnerability_noise(f_fine_khz)
    g_R = vulnerability_radiation(f_fine_khz)
    g_H = vulnerability_hypoxia(f_fine_khz)
    g_conv = (g_N * g_R * g_H) ** (1 / 3)

    ax.plot(f_fine_khz, g_N, color=PALETTE["orange"], lw=2.4,
            label="Noise (peak 4 kHz)")
    ax.plot(f_fine_khz, g_R, color=PALETTE["teal"], lw=2.4,
            label="Radiation (peak 6 kHz)")
    ax.plot(f_fine_khz, g_H, color=PALETTE["blue"], lw=2.4,
            label="Hypoxia (peak 3 kHz)")
    ax.plot(f_fine_khz, g_conv, color=PALETTE["red"], lw=3.0,
            label="Convergence (geo. mean)")
    ax.fill_between(f_fine_khz, 0, g_conv, color=PALETTE["red"], alpha=0.12)

    ax.axvspan(3.0, 6.0, color=PALETTE["olive"], alpha=0.20, zorder=0)

    ax.set_xscale("log")
    ax.set_xticks([0.5, 1, 2, 3, 4, 6, 8, 12])
    ax.set_xticklabels(["0.5", "1", "2", "3", "4", "6", "8", "12"])
    ax.set_xlim(0.5, 12)
    ax.set_xlabel("Frequency (kHz)")
    ax.set_ylabel("Normalized vulnerability  g(f)")
    ax.set_ylim(0, 1.1)
    ax.legend(loc="lower right", frameon=True, framealpha=0.95,
              edgecolor="#333333", facecolor="white")
    ax.text(0.50, 1.008, "TSC region (Kwak & Kwak 2020): 3, 4, or 6 kHz",
            transform=ax.transAxes, ha="center", va="bottom",
            fontsize=11,
            bbox=dict(facecolor="white", edgecolor=PALETTE["olive"],
                      boxstyle="round,pad=0.35", linewidth=1.0))

    plt.savefig(out_path)
    plt.close(fig)
    print(f"  saved {out_path.name}")


# ============================================================================
# 7) Figure 3 -- CCSI architecture + synthetic dataset distributions
# ============================================================================

def fig03_ccsi_architecture(df: 'pd.DataFrame', out_path: Path) -> None:
    """
    Fig 3.  CCSI model architecture and synthetic dataset characterisation.
    (A) Schematic flow: inputs -> physics dose proxies -> vulnerability
        kernels -> weighted sum -> CCSI(f).
    (B) Marginal distributions of the four primary input features
        (altitude, Rc, HCP, cabin noise) with kernel-density overlay.
    (C) Frequency-resolved CCSI output distribution (boxplot per band)
        showing the 3-6 kHz peak and band-to-band variance.
```

```
"""
from matplotlib.patches import FancyBboxPatch, FancyArrowPatch

fig, axes = plt.subplots(1, 3, figsize=(20, 10),
                         gridspec_kw=dict(wspace=0.38, hspace=0.55))

# ---------- Panel A: Architecture schematic (clean redesign) ----------
ax = axes[0]
style_axis(ax, title="CCSI model architecture")
add_panel_label(ax, "(A)")
ax.set_xlim(0, 12)
ax.set_ylim(0, 11)
ax.set_xticks([])
ax.set_yticks([])
for spine in ("left", "bottom"):
    ax.spines[spine].set_visible(False)
ax.grid(False)

def box(x, y, w, h, text, fc, ec="#333333", fontsize=11, weight="normal"):
    patch = FancyBboxPatch((x - w / 2, y - h / 2), w, h,
                           boxstyle="round,pad=0.05",
                           facecolor=fc, edgecolor=ec, linewidth=1.0)
    ax.add_patch(patch)
    ax.text(x, y, text, ha="center", va="center",
            fontsize=fontsize, fontweight=weight, color="#222222")

def arrow(x1, y1, x2, y2):
    ar = FancyArrowPatch((x1, y1), (x2, y2),
                         arrowstyle="->", mutation_scale=12,
                         linewidth=1.0, color=PALETTE["grey"])
    ax.add_patch(ar)

# Column header band
ax.text(1.65, 9.85, "INPUTS", ha="center", va="bottom",
        fontsize=11, fontweight="bold", color="#444444")
ax.text(5.75, 9.85, "PHYSICS  DOSE", ha="center", va="bottom",
        fontsize=11, fontweight="bold", color="#444444")
ax.text(9.95, 9.85, "VULNERABILITY", ha="center", va="bottom",
        fontsize=11, fontweight="bold", color="#444444")

# Inputs (left column)
in_x, in_w = 1.65, 2.9
for y, label in zip([9.0, 8.0, 7.0, 6.0, 5.0],
                    ["altitude $h$", "latitude $R_c$",
                     "solar HCP", "flight hours $t$",
                     "cabin noise"]):
    box(in_x, y, in_w, 0.74, label, "#F5E8C9", fontsize=10)

# Physics dose proxies (middle column), two-line labels
pp_x, pp_w = 5.75, 3.7
box(pp_x, 8.2, pp_w, 1.15, "Radiation $D_R$\n(CARI-7 approx.)", "#E8C9B0", fontsize=9.5)
box(pp_x, 6.8, pp_w, 1.15, "Hypoxia $D_H$\n(alveolar gas eq.)", "#C9DDE6", fontsize=9.5)
box(pp_x, 5.4, pp_w, 1.15, "Noise $D_N$\n(A-weighted)", "#E8B9A0", fontsize=9.5)

# Frequency kernels (right column), two-line labels
kk_x, kk_w = 9.95, 2.9
box(kk_x, 8.2, kk_w, 1.15, "$g_R(f)$\npeak 6 kHz", "#E8C9B0", fontsize=9.5)
box(kk_x, 6.8, kk_w, 1.15, "$g_H(f)$\npeak 3 kHz", "#C9DDE6", fontsize=9.5)
box(kk_x, 5.4, kk_w, 1.15, "$g_N(f)$\npeak 4 kHz", "#E8B9A0", fontsize=9.5)

# Output (bottom, wide centered)
box(6.0, 2.5, 10.5, 1.5,
    r"$\mathbf{CCSI(f) = w_R\,D_R\,g_R(f) \,+\, w_H\,D_H\,g_H(f) \,+\, w_N\,D_N\,g_N(f)}$",
    "#EAB4AE", ec="#7A2A26", fontsize=10, weight="bold")
ax.text(6.0, 1.25, "frequency-resolved cochlear stress  (8 bands: 0.5-12 kHz)",
        ha="center", va="center", fontsize=11, color="#7A2A26")

# Arrows: inputs (right edge ~3.1) -> physics proxies (left edge ~3.9)
arrow(3.1, 9.0, 3.9, 8.2)
arrow(3.1, 8.0, 3.9, 8.2)
```

```
arrow(3.1, 7.0, 3.9, 8.2)
arrow(3.1, 9.0, 3.9, 6.8)
arrow(3.1, 6.0, 3.9, 5.4)
arrow(3.1, 5.0, 3.9, 5.4)

# Arrows: physics (right edge ~7.6) -> kernels (left edge ~8.5)
for y in [8.2, 6.8, 5.4]:
    arrow(7.6, y, 8.5, y)

# Arrows: kernels/physics -> output box
arrow(9.95, 4.83, 7.5, 3.3)
arrow(5.75, 4.83, 5.5, 3.3)

# ---------- Panel B: Input feature distributions ----------
ax = axes[1]
style_axis(ax, title="Synthetic dataset input distributions")
add_panel_label(ax, "(B)")
ax.set_xticks([])
ax.set_yticks([])
for spine in ("left", "bottom"):
    ax.spines[spine].set_visible(False)
ax.grid(False)

from mpl_toolkits.axes_grid1.inset_locator import inset_axes
features = [
    ("altitude_km",   "Altitude (km)",          PALETTE["red"],    (3, 12.2)),
    ("Rc_GV",         r"$R_c$ (GV)",            PALETTE["orange"], (0, 17.5)),
    ("HCP_MV",        "HCP (MV)",               PALETTE["teal"],   (250, 1160)),
    ("noise_dBA",     r"Noise LA$_{eq}$ (dBA)", PALETTE["blue"],   (70, 95)),
]
positions = [(0.04, 0.55, 0.42, 0.40),
             (0.54, 0.55, 0.42, 0.40),
             (0.04, 0.06, 0.42, 0.40),
             (0.54, 0.06, 0.42, 0.40)]
for (col, lbl, color, xlim), pos in zip(features, positions):
    sub = inset_axes(ax, width="100%", height="100%",
                     bbox_to_anchor=pos, bbox_transform=ax.transAxes,
                     borderpad=0)
    vals = df[col].values
    sub.hist(vals, bins=22, color=color, alpha=0.78,
             edgecolor="#333333", linewidth=0.4)
    sub.set_xlabel(lbl, fontsize=12)
    sub.set_ylabel("count", fontsize=11)
    sub.set_xlim(xlim)
    sub.tick_params(axis="both", labelsize=10)
    sub.spines["top"].set_visible(False)
    sub.spines["right"].set_visible(False)
    sub.grid(True, color=PALETTE["lightgrey"], ls="--", lw=0.5, alpha=0.7)
    sub.set_facecolor("white")
    sub.axvline(np.mean(vals), color="#333333", ls=":", lw=1.0)

# ---------- Panel C: CCSI output distribution per frequency band ----------
ax = axes[2]
style_axis(ax, title="CCSI output distribution by frequency band")
add_panel_label(ax, "(C)")

freqs = FREQ_BANDS_HZ
ccsi_cols = [f"CCSI_{int(f)}Hz" for f in freqs]
ccsi_data = [df[c].values for c in ccsi_cols]

bp = ax.boxplot(
    ccsi_data, positions=np.arange(len(freqs)),
    widths=0.55, patch_artist=True,
    medianprops=dict(color="#222222", linewidth=1.6),
    whiskerprops=dict(color="#444444", linewidth=1.0),
    capprops=dict(color="#444444", linewidth=1.0),
    flierprops=dict(marker="o", markerfacecolor=PALETTE["grey"],
                    markersize=3.5, alpha=0.5, markeredgecolor="none"),
)
for i, patch in enumerate(bp["boxes"]):
```

```
        if 3000 <= freqs[i] <= 6000:
            patch.set_facecolor(PALETTE["red"])
            patch.set_alpha(0.78)
        else:
            patch.set_facecolor(PALETTE["orange"])
            patch.set_alpha(0.70)
        patch.set_edgecolor("#333333")
        patch.set_linewidth(0.7)

    ax.set_xticks(np.arange(len(freqs)))
    ax.set_xticklabels([f"{int(f / 1000)}" if f >= 1000 else f"{f/1000:.1f}"
                        for f in freqs])
    ax.set_xlabel("Frequency band (kHz)")
    ax.set_ylabel("CCSI score (dimensionless)")
    band_left = list(freqs).index(3000) - 0.5
    band_right = list(freqs).index(6000) + 0.5
    ax.axvspan(band_left, band_right, color=PALETTE["red"], alpha=0.06)

    plt.savefig(out_path)
    plt.close(fig)
    print(f"  saved {out_path.name}")


# ==============================================================================
# 8) ML pipeline -- Random Forest / Gradient Boosting / Ridge per frequency band
# ==============================================================================

FEATURE_COLS = [
    "altitude_km", "Rc_GV", "HCP_MV", "flight_hours",
    "noise_dBA", "hypoxia_index", "dose_radiation_uSv_per_h",
]

FEATURE_LABELS = {
    "altitude_km":              r"altitude $h$",
    "Rc_GV":                    r"cutoff $R_c$",
    "HCP_MV":                   r"solar HCP",
    "flight_hours":             r"flight $t$",
    "noise_dBA":                r"noise $L_{Aeq}$",
    "hypoxia_index":            r"hypoxia $H$",
    "dose_radiation_uSv_per_h": r"dose $D_R$",
}


def train_ml_models(df: 'pd.DataFrame', seed: int = 42) -> dict:
    """
    Train RF / GB / Ridge for each of the 8 frequency bands.
    Returns dict[model_name][band_hz] = {r2, mae, importance}.
    """
    X_all = df[FEATURE_COLS].values
    scaler = StandardScaler().fit(X_all)
    X = scaler.transform(X_all)

    models = {
        "Random Forest":      RandomForestRegressor(n_estimators=100,
                                                    random_state=seed,
                                                    n_jobs=1),
        "Gradient Boosting":  GradientBoostingRegressor(n_estimators=100,
                                                        learning_rate=0.1,
                                                        random_state=seed),
        "Ridge":              Ridge(alpha=1.0, random_state=seed),
    }

    results = {name: {} for name in models}
    kf = KFold(n_splits=5, shuffle=True, random_state=seed)

    for band in FREQ_BANDS_HZ:
        col = f"CCSI_{int(band)}Hz"
        y = df[col].values

        for name, mdl in models.items():
```

```
            # Cross-val R^2 and MAE
            r2_scores = cross_val_score(mdl, X, y, cv=kf, scoring="r2",
                                        n_jobs=1)
            mae_scores = -cross_val_score(mdl, X, y, cv=kf,
                                          scoring="neg_mean_absolute_error",
                                          n_jobs=1)
            mdl.fit(X, y)
            if hasattr(mdl, "feature_importances_"):
                imp = mdl.feature_importances_.copy()
            else:
                imp = np.abs(mdl.coef_) / (np.abs(mdl.coef_).sum() + 1e-9)

            results[name][int(band)] = {
                "r2_mean":  float(np.mean(r2_scores)),
                "r2_std":   float(np.std(r2_scores)),
                "mae_mean": float(np.mean(mae_scores)),
                "mae_std":  float(np.std(mae_scores)),
                "importance": imp.tolist(),
            }
    return results


def fig04_ml_results(ml_results, out_path):
    fig, axes = plt.subplots(1, 3, figsize=(20, 10),
                             gridspec_kw=dict(wspace=0.40, hspace=0.55))

    model_names = list(ml_results.keys())
    short_names = {"Random Forest": "RF", "Gradient Boosting": "GB",
                   "Ridge": "Ridge"}
    model_colors = [PALETTE["red"], PALETTE["orange"], PALETTE["teal"]]

    # ---------- Panel A: R^2 + MAE per model (short labels) ----------
    ax = axes[0]
    style_axis(ax, title=r"Cross-validated $R^2$ and MAE per model")
    add_panel_label(ax, "(A)")

    r2_means, r2_stds, mae_means, mae_stds = [], [], [], []
    for name in model_names:
        r2_means.append(np.mean(
            [ml_results[name][int(b)]["r2_mean"] for b in FREQ_BANDS_HZ]))
        r2_stds.append(np.std(
            [ml_results[name][int(b)]["r2_mean"] for b in FREQ_BANDS_HZ]))
        mae_means.append(np.mean(
            [ml_results[name][int(b)]["mae_mean"] for b in FREQ_BANDS_HZ]))
        mae_stds.append(np.std(
            [ml_results[name][int(b)]["mae_mean"] for b in FREQ_BANDS_HZ]))

    xpos = np.arange(len(model_names))
    width = 0.35
    bars1 = ax.bar(xpos - width / 2, r2_means, width, yerr=r2_stds, capsize=5,
                   color=model_colors, edgecolor="#333333", linewidth=0.6,
                   label=r"$R^2$", error_kw=dict(ecolor="#444444", lw=1.0))
    # MAE on secondary y-axis
    ax2 = ax.twinx()
    ax2.spines["top"].set_visible(False)
    bars2 = ax2.bar(xpos + width / 2, mae_means, width, yerr=mae_stds,
                    capsize=5,
                    color=[PALETTE["olive"]] * 3, edgecolor="#333333",
                    linewidth=0.6, label="MAE",
                    error_kw=dict(ecolor="#444444", lw=1.0), alpha=0.85)

    for bar, v in zip(bars1, r2_means):
        ax.text(bar.get_x() + bar.get_width() / 2, bar.get_height() + 0.025,
                f"{v:.3f}", ha="center", va="bottom", fontsize=10,
                color="#333333")
    for bar, v in zip(bars2, mae_means):
        ax2.text(bar.get_x() + bar.get_width() / 2, bar.get_height() * 1.05,
                 f"{v:.3f}", ha="center", va="bottom", fontsize=10,
                 color="#5C4D2A")
```

```
ax.set_xticks(xpos)
ax.set_xticklabels([short_names[n] for n in model_names])
ax.set_ylabel(r"5-fold CV  $R^2$")
ax2.set_ylabel("Mean absolute error  (CCSI units)", color="#5C4D2A")
ax.set_ylim(0, 1.15)
ax2.set_ylim(0, max(mae_means) * 1.6 + 1e-6)
ax2.tick_params(axis="y", colors="#5C4D2A")

ax.text(0.50, 1.035, "All three models near $R^2$ = 0.98; RF / GB tied",
        transform=ax.transAxes, ha="center", va="bottom",
        fontsize=11,
        bbox=dict(facecolor="white", edgecolor="#333333",
                  boxstyle="round,pad=0.35", linewidth=0.8))

# ---------- Panel B: Feature importance donut (RF, 4 kHz) ----------
ax = axes[1]
add_panel_label(ax, "(B)")
ax.set_title("Feature importance -- RF at 4 kHz", pad=6, fontsize=16)
ax.set_facecolor("white")
ax.set_xticks([]); ax.set_yticks([])
for spine in ("left", "bottom"):
    ax.spines[spine].set_visible(False)
ax.grid(False)
ax.set_aspect("equal")
ax.set_xlim(-1.5, 1.5)
ax.set_ylim(-1.5, 1.5)

imp = np.array(ml_results["Random Forest"][4000]["importance"])
labels = [FEATURE_LABELS[c] for c in FEATURE_COLS]
order = np.argsort(imp)[::-1]
imp_sorted = imp[order]
labels_sorted = [labels[i] for i in order]

# Build donut data: keep slices >= 1%, group remainder as "other"
threshold = 0.01
donut_vals, donut_lbls, donut_cols = [], [], []
palette_seq = [PALETTE["red"], PALETTE["orange"], "#D17B3A",
               PALETTE["teal"], PALETTE["blue"], PALETTE["olive"]]
other_total = 0.0
other_details = []
for v, lbl, c in zip(imp_sorted, labels_sorted, palette_seq):
    if v >= threshold:
        donut_vals.append(v)
        donut_lbls.append(lbl)
        donut_cols.append(c)
    else:
        other_total += v
        other_details.append((lbl, v))
if other_total > 0:
    donut_vals.append(other_total)
    donut_lbls.append(f"other  ({len(other_details)} feats)")
    donut_cols.append(PALETTE["lightgrey"])

# Donut shifted up; legend will sit BELOW the donut, inside the panel
wedges, _ = ax.pie(
    donut_vals, labels=None, colors=donut_cols,
    startangle=90, counterclock=False,
    radius=0.85,
    center=(0.0, 0.40),
    wedgeprops=dict(width=0.38, edgecolor="#333333", linewidth=0.9),
)

# Centre annotation
ax.text(0, 0.45, "4 kHz", ha="center", va="center",
        fontsize=16, fontweight="bold", color="#333333")
ax.text(0, 0.27, "CCSI feature\ncontribution", ha="center", va="center",
        fontsize=10, color="#555555")

# Legend BELOW donut -- multi-column, stays inside panel (no bleed)
legend_entries = [f"{lbl}:  {v * 100:5.2f}%"
```

```
                 for lbl, v in zip(donut_lbls, donut_vals)]
leg = ax.legend(wedges, legend_entries,
                loc="lower center", bbox_to_anchor=(0.5, -0.03),
                frameon=True, edgecolor="#333333", framealpha=0.95,
                facecolor="white", fontsize=10,
                handlelength=1.3, borderpad=0.5,
                ncol=2, columnspacing=1.2, labelspacing=0.35)

ax.text(0.50, 1.035,
        "Flight-hours dominant (CCSI scales linearly with $t$)",
        transform=ax.transAxes, ha="center", va="bottom",
        fontsize=11,
        bbox=dict(facecolor="white", edgecolor=PALETTE["red"],
                  boxstyle="round,pad=0.35", linewidth=1.0))

# ---------- Panel C: Feature importance heatmap (7 features x 8 bands) ----------
ax = axes[2]
style_axis(ax, title="RF importance heatmap (features $\\times$ bands)")
add_panel_label(ax, "(C)")

# Build matrix: rows = features (ordered), cols = freq bands
band_order = list(FREQ_BANDS_HZ)
imp_matrix = np.zeros((len(FEATURE_COLS), len(band_order)))
for j, band in enumerate(band_order):
    imp_matrix[:, j] = np.array(
        ml_results["Random Forest"][int(band)]["importance"])

# Row-normalise so each feature's pattern across bands is visible
row_max = imp_matrix.max(axis=1, keepdims=True)
row_max[row_max < 1e-9] = 1.0
imp_rownorm = imp_matrix / row_max

# Custom warming-earthtone cmap (white -> orange -> red)
from matplotlib.colors import LinearSegmentedColormap
warm_cmap = LinearSegmentedColormap.from_list(
    "warm_earth", ["#FFFFFF", "#F5E8C9", "#E8943A", "#C94F4A"], N=256)

im = ax.imshow(imp_rownorm, aspect="auto", cmap=warm_cmap,
               vmin=0, vmax=1)

ax.set_xticks(np.arange(len(band_order)))
ax.set_xticklabels([f"{int(f / 1000)}" if f >= 1000 else f"{f/1000:.1f}"
                    for f in band_order])
ax.set_yticks(np.arange(len(FEATURE_COLS)))
ax.set_yticklabels([FEATURE_LABELS[c] for c in FEATURE_COLS])
ax.set_xlabel("Frequency band (kHz)")
ax.set_ylabel("Input feature")
ax.set_axisbelow(True)
ax.grid(False)

# Annotate each cell with row-normalised value
for i in range(len(FEATURE_COLS)):
    for j in range(len(band_order)):
        val = imp_rownorm[i, j]
        color = "#FFFFFF" if val > 0.55 else "#333333"
        ax.text(j, i, f"{val:.2f}", ha="center", va="center",
                fontsize=9, color=color)

# 3-6 kHz convergence column band overlay (subtle)
j_lo = band_order.index(3000) - 0.5
j_hi = band_order.index(6000) + 0.5
ax.axvline(j_lo, color=PALETTE["red"], lw=1.0, ls="--", alpha=0.6)
ax.axvline(j_hi, color=PALETTE["red"], lw=1.0, ls="--", alpha=0.6)

cbar = fig.colorbar(im, ax=ax, fraction=0.045, pad=0.04,
                    ticks=[0, 0.5, 1])
cbar.ax.tick_params(labelsize=10)
cbar.set_label("row-normalised importance", fontsize=11)

ax.text(0.50, 1.035,
```

```
            "Per-feature band patterns: $g_R/g_H/g_N$ activate distinct bands",
            transform=ax.transAxes, ha="center", va="bottom",
            fontsize=11,
            bbox=dict(facecolor="white", edgecolor=PALETTE["red"],
                      boxstyle="round,pad=0.35", linewidth=1.0))

    plt.savefig(out_path)
    plt.close(fig)
    print(f"  saved {out_path.name}")


# ==============================================================================
# 9) Figure 5 -- TSC region prioritization (clinical translation)
# ==============================================================================

# Four typical aircrew operational profiles
AIRCREW_PROFILES = [
    {"name": "Polar long-haul",
     "altitude_km": 11.0, "Rc_GV": 1.0,  "HCP_MV": 600.0,
     "flight_hours": 10.0, "noise_dBA": 78.0,
     "desc": "FL360, Svalbard route, wide-body"},
    {"name": "Mid-lat long-haul",
     "altitude_km": 11.0, "Rc_GV": 9.96, "HCP_MV": 1141.0,
     "flight_hours": 8.0,  "noise_dBA": 78.0,
     "desc": "FL360, Korea/Asia, wide-body"},
    {"name": "Domestic short-haul",
     "altitude_km": 8.5,  "Rc_GV": 9.96, "HCP_MV": 1141.0,
     "flight_hours": 1.5,  "noise_dBA": 80.0,
     "desc": "FL280, narrow-body, 1.5 h"},
    {"name": "Military fighter / rotary",
     "altitude_km": 6.0,  "Rc_GV": 9.96, "HCP_MV": 1141.0,
     "flight_hours": 1.5,  "noise_dBA": 92.0,
     "desc": "FL200, high-noise, short missions"},
]


def fig05_tsc_prioritization(out_path: Path) -> None:
    """
    Fig 5.  TSC region prioritization for representative aircrew profiles.
    (A) CCSI heatmap (4 profiles x 8 frequency bands).
    (B) Per-profile CCSI(f) line overlay.
    (C) Recommended TSC region per profile (argmax in 3/4/6 kHz Kwak set).
    """
    fig, axes = plt.subplots(1, 3, figsize=(20, 10),
                             gridspec_kw=dict(wspace=0.42, hspace=0.55))

    n_prof = len(AIRCREW_PROFILES)
    profile_ccsi = np.zeros((n_prof, len(FREQ_BANDS_KHZ)))
    for i, p in enumerate(AIRCREW_PROFILES):
        ccsi_i = compute_CCSI(
            np.array([p["altitude_km"]]),
            np.array([p["flight_hours"]]),
            np.array([p["Rc_GV"]]),
            np.array([p["HCP_MV"]]),
            np.array([p["noise_dBA"]]),
        )
        profile_ccsi[i, :] = ccsi_i[0, :]

    profile_names = [p["name"] for p in AIRCREW_PROFILES]
    profile_colors = [PALETTE["red"], PALETTE["orange"],
                      PALETTE["teal"], PALETTE["blue"]]

    ax = axes[0]
    add_panel_label(ax, "(A)")
    ax.set_title(r"CCSI heatmap (profile $\times$ frequency band)",
                 pad=28, fontsize=13.5, fontweight="bold")
    ax.set_facecolor("white")
    ax.grid(False)

    from matplotlib.colors import LinearSegmentedColormap
```

```
warm_cmap = LinearSegmentedColormap.from_list(
    "warm_earth", ["#FFFFFF", "#F5E8C9", "#E8943A", "#C94F4A"], N=256)
vmax = float(profile_ccsi.max() * 1.02)

im = ax.imshow(profile_ccsi, aspect="auto", cmap=warm_cmap,
               vmin=0, vmax=vmax)
ax.set_xticks(np.arange(len(FREQ_BANDS_HZ)))
ax.set_xticklabels([f"{int(f / 1000)}" if f >= 1000 else f"{f/1000:.1f}"
                    for f in FREQ_BANDS_HZ])
ax.set_yticks(np.arange(n_prof))
ax.set_yticklabels(profile_names)
ax.set_xlabel("Frequency band (kHz)")

for i in range(n_prof):
    for j in range(len(FREQ_BANDS_HZ)):
        val = profile_ccsi[i, j]
        color = "#FFFFFF" if val / vmax > 0.55 else "#333333"
        ax.text(j, i, f"{val:.2f}", ha="center", va="center",
                fontsize=9, color=color)

band_lo = list(FREQ_BANDS_HZ).index(3000) - 0.5
band_hi = list(FREQ_BANDS_HZ).index(6000) + 0.5
ax.axvline(band_lo, color=PALETTE["red"], lw=1.0, ls="--", alpha=0.7)
ax.axvline(band_hi, color=PALETTE["red"], lw=1.0, ls="--", alpha=0.7)

cbar = fig.colorbar(im, ax=ax, fraction=0.045, pad=0.04)
cbar.set_label("CCSI score", fontsize=11)
cbar.ax.tick_params(labelsize=10)

ax.text(0.50, 1.008, "Dashed: Kwak TSC region (3-6 kHz)",
        transform=ax.transAxes, ha="center", va="bottom",
        fontsize=11,
        bbox=dict(facecolor="white", edgecolor=PALETTE["red"],
                  boxstyle="round,pad=0.35", linewidth=1.0))

ax = axes[1]
style_axis(ax)
ax.set_title("CCSI vs frequency, per aircrew profile",
             fontsize=13.5, fontweight="bold", pad=28)
add_panel_label(ax, "(B)")

for i, p in enumerate(AIRCREW_PROFILES):
    ax.plot(FREQ_BANDS_KHZ, profile_ccsi[i, :],
            color=profile_colors[i], lw=2.4, marker="o", markersize=7,
            label=p["name"])

ax.axvspan(3.0, 6.0, color=PALETTE["olive"], alpha=0.18, zorder=0)
ax.set_xscale("log")
ax.set_xticks([0.5, 1, 2, 3, 4, 6, 8, 12])
ax.set_xticklabels(["0.5", "1", "2", "3", "4", "6", "8", "12"])
ax.set_xlim(0.45, 13)
ax.set_xlabel("Frequency (kHz)")
ax.set_ylabel("CCSI score")
ax.set_ylim(0, 0.55)
_wrap = {"Polar long-haul": "Polar\nlong-haul",
         "Mid-lat long-haul": "Mid-lat\nlong-haul",
         "Domestic short-haul": "Domestic\nshort-haul",
         "Military fighter / rotary": "Military\nfighter/rotary"}
_h, _l = ax.get_legend_handles_labels()
ax.legend(_h, [_wrap.get(x, x) for x in _l],
          loc="upper left", frameon=True, framealpha=0.95,
          edgecolor="#333333", facecolor="white", fontsize=9.5,
          handlelength=1.4, labelspacing=0.7, borderpad=0.5)

ax.text(0.50, 1.008,
        "Olive band: Kwak TSC region; all profiles peak inside",
        transform=ax.transAxes, ha="center", va="bottom",
        fontsize=11,
        bbox=dict(facecolor="white", edgecolor=PALETTE["olive"],
                  boxstyle="round,pad=0.35", linewidth=1.0))
```

```
    ax = axes[2]
    style_axis(ax)
    ax.set_title("Recommended TSC region per profile  (Kwak 3/4/6 kHz)",
                 fontsize=13.5, fontweight="bold", pad=28)
    add_panel_label(ax, "(C)")

    tsc_options_hz = np.array([3000, 4000, 6000])
    tsc_idx = [list(FREQ_BANDS_HZ).index(f) for f in tsc_options_hz]
    tsc_subset = profile_ccsi[:, tsc_idx]
    recommended_idx = np.argmax(tsc_subset, axis=1)
    recommended_freq = tsc_options_hz[recommended_idx] / 1000.0

    xpos = np.arange(n_prof)
    width = 0.25
    for k, (band_hz, lbl) in enumerate(zip(tsc_options_hz,
                                           ["3 kHz", "4 kHz", "6 kHz"])):
        vals = tsc_subset[:, k]
        is_rec = (recommended_freq == band_hz / 1000.0)
        colors_k = [PALETTE["red"] if r else "#E8C9B0" for r in is_rec]
        ax.bar(xpos + (k - 1) * width, vals, width,
               color=colors_k, edgecolor="#333333", linewidth=0.6,
               label=lbl)
        for x_off, v in zip(xpos + (k - 1) * width, vals):
            ax.text(x_off, v + 0.01 * vmax,
                    f"{v:.2f}", ha="center", va="bottom",
                    fontsize=9, color="#333333")

    ax.set_xticks(xpos)
    ax.set_xticklabels([p["name"] for p in AIRCREW_PROFILES],
                       rotation=15, ha="right")
    ax.set_ylabel("CCSI at TSC option (3/4/6 kHz)")
    ax.set_ylim(0, 0.64)
    ax.legend(loc="upper left", frameon=True, framealpha=0.95,
              edgecolor="#333333", facecolor="white", fontsize=11,
              title="TSC option", title_fontsize=11)

    ax.text(0.50, 1.008,
            "Filled red bar = recommended TSC region for that profile",
            transform=ax.transAxes, ha="center", va="bottom",
            fontsize=11,
            bbox=dict(facecolor="white", edgecolor=PALETTE["red"],
                      boxstyle="round,pad=0.35", linewidth=1.0))

    plt.savefig(out_path)
    plt.close(fig)
    print(f"  saved {out_path.name}")


def sensitivity_analysis(out_path: Path,
                         json_path: Path) -> None:
    """
    Sensitivity analysis over 6 weight scenarios x 4 aircrew profiles.
    Computes CCSI on the FULL discrete audiometric grid
    F = {0.5, 1, 2, 3, 4, 6, 8, 12} kHz and reports the GLOBAL argmax
    (not restricted to {3,4,6} kHz) to provide a non-tautological
    robustness check for the convergence hypothesis.
    """
    scenarios = [
        ("Default (noise-heavy prior)", 0.45, 0.20, 0.35),
        ("Balanced",                    0.40, 0.30, 0.30),
        ("Noise-extreme",               0.50, 0.10, 0.40),
        ("Hypoxia-extreme",             0.30, 0.50, 0.20),
        ("Radiation-extreme",           0.30, 0.20, 0.50),
        ("Radiation-excluded (wR=0)",   0.69, 0.31, 0.00),
    ]
    grid_khz = FREQ_BANDS_KHZ            # full audiometric grid
    grid_labels = ["0.5", "1", "2", "3", "4", "6", "8", "12"]
    TSC_set = {3.0, 4.0, 6.0}
```

```
    rows = []
    summary = {"grid_khz": list(grid_khz), "scenarios": []}
    inside_TSC = 0
    total = 0
    for sc_name, wN, wH, wR in scenarios:
        sc_block = {"name": sc_name, "weights": [wN, wH, wR],
                    "profiles": []}
        for prof in AIRCREW_PROFILES:
            ccsi = compute_CCSI(
                altitude_km=np.array([prof["altitude_km"]]),
                flight_hours=np.array([prof["flight_hours"]]),
                Rc_GV=np.array([prof["Rc_GV"]]),
                HCP_MV=np.array([prof["HCP_MV"]]),
                noise_dBA=np.array([prof["noise_dBA"]]),
                weights=CCSIWeights(w_R=wR, w_N=wN, w_H=wH),
                freq_khz=grid_khz,
            )[0, :]
            global_arg_idx = int(np.argmax(ccsi))
            global_arg_kHz = float(grid_khz[global_arg_idx])
            row = {
                "weight_set": sc_name,
                "w_N": wN, "w_H": wH, "w_R": wR,
                "profile": prof["name"],
                "global_argmax_kHz": global_arg_kHz,
                "argmax_in_TSC": global_arg_kHz in TSC_set,
            }
            for lab, val in zip(grid_labels, ccsi):
                row[f"CCSI_{lab}k"] = round(float(val), 4)
            rows.append(row)
            sc_block["profiles"].append(
                {"profile": prof["name"],
                 "global_argmax_kHz": global_arg_kHz,
                 "ccsi": [round(float(v), 4) for v in ccsi]}
            )
            total += 1
            if global_arg_kHz in TSC_set:
                inside_TSC += 1
        summary["scenarios"].append(sc_block)

    summary["total_cells"] = total
    summary["cells_argmax_in_TSC"] = inside_TSC
    summary["fraction_in_TSC"] = inside_TSC / total

    # Save CSV
    cols = (["weight_set", "w_N", "w_H", "w_R", "profile",
             "global_argmax_kHz", "argmax_in_TSC"]
            + [f"CCSI_{lab}k" for lab in grid_labels])
    pd.DataFrame(rows).to_csv(out_path, index=False, columns=cols)
    with open(json_path, "w") as f:
        json.dump(summary, f, indent=2)
    print(f"  sensitivity: {inside_TSC}/{total} cells argmax in {{3,4,6}} kHz")
    print(f"  saved {out_path.name} and {json_path.name}")


def main(out_dir="."):
    out = Path(out_dir)
    out.mkdir(parents=True, exist_ok=True)

    print("[1/7] Generating synthetic dataset (n=500)...")
    df = generate_synthetic_dataset(n=500, seed=42)
    df.to_csv(out / "synthetic_dataset.csv", index=False)
    print(f"  saved synthetic_dataset.csv shape={df.shape}")

    print("[2/7] Generating Figure 1 -- Multi-hazard overview...")
    fig01_multihazard(df, out / "fig_01_multihazard_overview.png")

    print("[3/7] Generating Figure 2 -- 3-6 kHz convergence...")
    fig02_frequency_convergence(out / "fig_02_frequency_convergence.png")

    print("[4/7] Generating Figure 3 -- CCSI architecture + dataset...")
```

```
    fig03_ccsi_architecture(df, out / "fig_03_ccsi_architecture.png")

    print("[5/7] Training ML ensemble (RF/GB/Ridge x 8 bands)...")
    ml_results = train_ml_models(df, seed=42)
    with open(out / "ml_results.json", "w") as f:
        json.dump(ml_results, f, indent=2)
    print(f"  saved ml_results.json")

    print("[6/7] Generating Figure 4 -- ML performance + importance...")
    fig04_ml_results(ml_results, out / "fig_04_ml_results.png")

    print("[7/7] Generating Figure 5 -- TSC prioritization...")
    fig05_tsc_prioritization(out / "fig_05_tsc_prioritization.png")

    print("[+]   Running full-grid sensitivity analysis...")
    sensitivity_analysis(out / "sensitivity_table.csv",
                         out / "sensitivity_results.json")

    print("Done. All 5 figures + sensitivity table generated.")


if __name__ == "__main__":
    import sys
    out = sys.argv[1] if len(sys.argv) > 1 else "."
    main(out)
```

S3.2 `null_test.py` — anti-tautology Monte-Carlo

```
"""
Anti-tautology Monte Carlo for the convergence hypothesis (H1).
Tests whether argmax of the geometric-mean convergence kernel landing
inside the clinical TSC set {3,4,6} kHz is (a) robust to mechanistic
parameter uncertainty and (b) NOT automatic for arbitrary kernels.
"""
import numpy as np

F = np.array([0.5,1,2,3,4,6,8,12])          # discrete audiometric grid
TSC = {3.0,4.0,6.0}

def conv_argmax(muN,muH,muR,sN,sH,sR):
    def g(f,mu,s): return np.exp(-0.5*(np.log2(f/mu)/s)**2)
    c = (g(F,muN,sN)*g(F,muH,sH)*g(F,muR,sR))**(1/3)
    return F[int(np.argmax(c))]

rng = np.random.default_rng(42)
N = 200000

# ---- Scenario A: mechanistic uncertainty (literature-bounded) ----
muN = rng.uniform(3.5,4.5,N)   # noise notch ~4 kHz
muH = rng.uniform(2.5,3.5,N)   # hypoxia 2-4 kHz band, center ~3
muR = rng.uniform(5.0,7.0,N)   # radiation high-freq bias ~6
sN  = rng.uniform(0.7,1.1,N); sH=rng.uniform(0.7,1.1,N); sR=rng.uniform(0.7,1.1,N)
amA = np.array([conv_argmax(*p) for p in zip(muN,muH,muR,sN,sH,sR)])
pA = np.mean([a in TSC for a in amA]); p4A = np.mean(amA==4.0)

# ---- Scenario B: unconstrained null (peaks log-uniform over 1-8 kHz) ----
lo,hi = np.log2(1.0), np.log2(8.0)
mN = 2**rng.uniform(lo,hi,N); mH = 2**rng.uniform(lo,hi,N); mR = 2**rng.uniform(lo,hi,N)
sN2=rng.uniform(0.7,1.1,N); sH2=rng.uniform(0.7,1.1,N); sR2=rng.uniform(0.7,1.1,N)
amB = np.array([conv_argmax(*p) for p in zip(mN,mH,mR,sN2,sH2,sR2)])
pB = np.mean([a in TSC for a in amB]); p4B = np.mean(amB==4.0)

print(f"N = {N}")
print(f"[A] mechanistic uncertainty : P(argmax in TSC) = {pA*100:.1f}%  | P(argmax=4kHz) = {p4A*100:.1f}%")
print(f"[B] unconstrained null      : P(argmax in TSC) = {pB*100:.1f}%  | P(argmax=4kHz) = {p4B*100:.1f}%")
import collections
print("[A] argmax dist:", {float(k):round(v/N,3) for k,v in sorted(collections.Counter(amA).items())})
print("[B] argmax dist:", {float(k):round(v/N,3) for k,v in sorted(collections.Counter(amB).items())})
```

S4 RELEASED DATA FILES

Table S3: Released data and code artefacts (Zenodo; DOI on acceptance).

File	Contents
analysis_main.py	CCSI implementation, dose proxies, kernels, dataset generator, ML pipeline, sensitivity, figures.
null_test.py	Anti-tautology Monte-Carlo (Section S1.3).
synthetic_dataset.csv	$n = 500$ aircrew profiles with per-band CCSI labels.
ml_results.json	5-fold CV R^2/MAE and feature importances per band.
sensitivity_results.json	Full 5×4 weight $\times$ profile CCSI output.
sensitivity_table.csv	Per-cell CCSI values, all eight bands (main Table 2).
null_test_results.json	Monte-Carlo summary statistics (Table S2).
